# Genome sequences and comparative genomics of two *Lactobacillus ruminis* strains from the bovine and human intestinal tracts

**DOI:** 10.1186/1475-2859-10-S1-S13

**Published:** 2011-08-30

**Authors:** Brian M Forde, B Anne Neville, Michelle M O’ Donnell, E Riboulet-Bisson, M J Claesson, Avril Coghlan, R Paul Ross, Paul W O’ Toole

**Affiliations:** 1 Department Microbiology, University College Cork, Ireland; 2 Teagasc Food Research Centre, Moorepark, Fermoy, Co. Cork, Ireland

## Abstract

**Background:**

The genus *Lactobacillus* is characterized by an extraordinary degree of phenotypic and genotypic diversity, which recent genomic analyses have further highlighted. However, the choice of species for sequencing has been non-random and unequal in distribution, with only a single representative genome from the *L. salivarius* clade available to date. Furthermore, there is no data to facilitate a functional genomic analysis of motility in the lactobacilli, a trait that is restricted to the *L. salivarius* clade.

**Results:**

The 2.06 Mb genome of the bovine isolate *Lactobacillus ruminis* ATCC 27782 comprises a single circular chromosome, and has a G+C content of 44.4%. *In silico* analysis identified 1901 coding sequences, including genes for a pediocin-like bacteriocin, a single large exopolysaccharide-related cluster, two sortase enzymes, two CRISPR loci and numerous IS elements and pseudogenes. A cluster of genes related to a putative pilin was identified, and shown to be transcribed *in vitro*. A high quality draft assembly of the genome of a second *L. ruminis* strain, ATCC 25644 isolated from humans, suggested a slightly larger genome of 2.138 Mb, that exhibited a high degree of synteny with the ATCC 27782 genome. In contrast, comparative analysis of *L. ruminis* and *L. salivarius* identified a lack of long-range synteny between these closely related species. Comparison of the *L. salivarius* clade core proteins with those of nine other *Lactobacillus* species distributed across 4 major phylogenetic groups identified the set of shared proteins, and proteins unique to each group.

**Conclusions:**

The genome of *L. ruminis* provides a comparative tool for directing functional analyses of other members of the *L. salivarius* clade, and it increases understanding of the divergence of this distinct *Lactobacillus* lineage from other commensal lactobacilli. The genome sequence provides a definitive resource to facilitate investigation of the genetics, biochemistry and host interactions of these motile intestinal lactobacilli*.*

## Background

The lactic acid bacteria (LAB) are low G+C, Gram-positive bacteria that produce lactic acid through the fermentation of hexose sugars [1]. The LAB are not a monophyletic group, but rather a pragmatic phenotypic division encompassing 13 genera. The largest of these is the genus *Lactobacillus*, with over 171 currently recognized species [2]. The lactobacilli are considered a subdominant element in the human gastrointestinal tract (GIT) and have been extensively studied for both their industrial application and health benefits [3]. The genus *Lactobacillus* is highly diverse [4]. On the basis of phylogenetic markers such as the 16S rRNA [5] or the *groEL* gene [6], clades or clusters of species have been defined within the genus *Lactobacillus*. In the most recent comprehensive description of this genus, twelve *Lactobacillus* and two *Pediococcus* clades were proposed [5]. The process of assigning species to clades within a larger genus is not novel, and cladistics has formed an integral part of many *Lactobacillus* phylogenetic analyses [4,5,7-10]. As more species are identified, a clearer resolution of the clades emerges. For example, the *L. plantarum* group originally included twelve species [8], but has since undergone significant reclassification, and now contains only three species, namely *L. plantarum*, *L. paraplantarum* and *L. pentosus*[*5*]. Furthermore, the *L. buchneri* group that was a major clade in early *Lactobacillus* phylogenies [8] has since been revised, and robust divisions within the group are evident [5]

The *L. acidophilus* group [4], formerly known as the *L. delbrueckii* group [11], is one of the largest *Lactobacillus* clades. It harbours the “*L. acidophilus* complex”, a cluster of several species including *L. acidophilus*, *L. amylovorus*, *L. crispatus*, *L. gallinarum*, *L. gasseri*, *L. helveticus* and *L. johnsonii*[12-14] that were mistakenly identified as *L. acidophilus* strains upon their original isolation [13,15]. Members of this clade have been isolated from humans and environmental sources, and represent some of the best characterised lactobacilli. Similarly, the *L. salivarius* and *L. reuteri* clades were named after the best characterised of their member species and may be considered as major phylogenetic units within the genus *Lactobacillus*. The *L. reuteri* clade includes member species that were isolated either from humans (*L. antri; L. coleohominis; L. gastricus; L. oris; L. vaginalis*), animals (*L. reuteri*) or birds (*L. ingluviei*) or from foods such as rye-bran fermentations (*L. frumenti*) and sourdough (*L. panis*; *L. pontis* and *L. secaliphilus*) [2]. Likewise, the species comprising the *L. salivarius* clade have been isolated from vertebrate intestine/faeces, soil, water and plants or food [16]. This clade includes *L. ruminis* which is phylogenetically close to *L. salivarius*[*11*] and which shares the same ecological niche [17-19].

Application of genomic technologies has been very beneficial for understanding the biology of commensal lactobacilli [20]. The full genomes of 14 *Lactobacillus* species have been sequenced and published [18,21-31] and 140 *Lactobacillus *sequencing projects are on-going [32]. There is a bias towards the analysis of species that are phylogenetically close to *L. acidophilus*: of the 14 *Lactobacillus* genomes currently available, 6 are from the *L. acidophilus* complex. Until recently, only one genome from a member of the *L. salivarius* clade had been fully sequenced [30]. Additionally, while the development of next generation sequencing technologies has led to a near exponential increase in the number of sequenced bacterial genomes, the majority of these genomes remain at low quality level, have been assembled and scaffolded without human intervention, contain numerous sequence gaps and are poorly annotated. As a consequence these draft genome sequences are often unsuitable for whole genome comparative analysis, particularly where the emphasis is on synteny, operon structure, or plasmid configuration.

*Lactobacillus ruminis* was first isolated from the faeces of humans in 1960 [33] and subsequently from the bovine rumen [17]. *L. ruminis* has been identified as one of 17 species of lactobacilli which are routinely isolated from the faeces of humans [19], cattle [34] and pigs [35] and is considered to be a member of the autochthonous microbiota in the gastrointestinal tract (GIT) [18,19]. *L. ruminis* is unusual among the lactobacilli as it is one of only 14 members of this genus to be characterised as being motile [36]. As well as being motile, *L. ruminis* is of interest because the immunomodulatory characteristics of this species, specifically its ability to stimulate tumour necrosis factor (TNF) and nuclear-factor κB (NF- κB) production in monocytes [37], has identified *L. ruminis* as a candidate probiotic. In this study, we determined the genome sequence of *Lactobacillus ruminis* ATCC 27782 (a motile strain isolated from cows), representing the first genome sequence of a motile *Lactobacillus* and the second completely finished [38] genome from a member of the *L. salivarius* clade.

## Results and discussion

### General genome features

**Figure 1:**
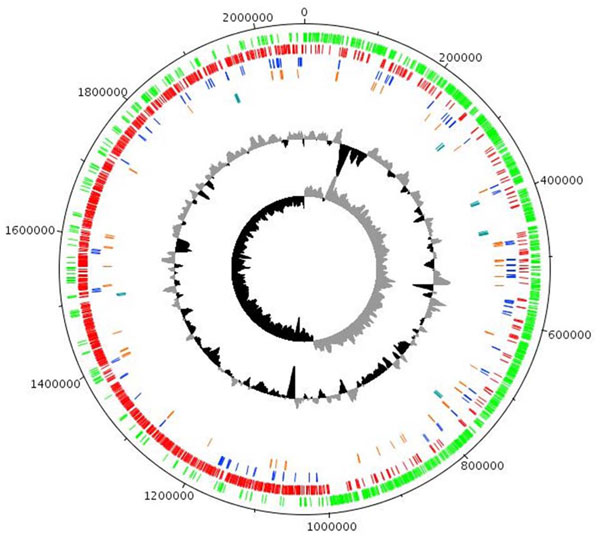
**Genome atlas of *L. ruminis* ATCC 27782.** This graphical representation of the genome was generated using DNAPLOTTER. From outside to inside: *L. ruminis* genes on the forward strand (green); *L. ruminis* genes on the reverse strand (red); pseudogenes (blue); insertion sequence elements (orange); ribosomal RNA genes (Cyan); GC% (Black below mean and grey above mean); GC skew.

The genome of *Lactobacillus ruminis* ATCC 27782 consists of a singular circular chromosome of 2,066,657 bp with an average G+C content of 44.4% (Table 1). Bioinformatic analysis of the genome identified 1901 coding regions, representing a coding density of 80.5%, and with an average gene length of 875 bp. Biological functions could be assigned to 1417 (72.2%) of the predicted proteins. The remaining 473 (23.9%) were found to be homologous to conserved hypothetical proteins in other species or had no match to any known protein. The GC% map of the genome of *L. ruminis* ATCC 27782 (Figure 1) identifies several regions with significantly deviating GC content. The first and largest of these regions (100,290 to 166,099 bp) corresponds to an exopolysaccharide biosynthesis locus (see below). The second region (563,932 to 574,637 bp) is flanked by integrases and contains a number of hypothetical proteins. Also located in this region are a recombinase and a DNA cytosine-5-methyltransferase, both of which are classified as pseudogenes due to frameshifts. The third region (1,068,439 to 1,077,247 bp) corresponds to the *cas* genes of CRISPR region 2 (see below).

**Table 1:** Comparison of the major genomic features of *L. ruminis* ATCC 27782, *L. ruminis* ATCC 25644, and *L. salivarius* UCC118. Figures for ATCC 25644 are estimates based on the draft assembly and automated annotation, and pseudogenes were not predicted due to low quality regions and sequence gaps. Numbers in parentheses for *L. salivarius* UCC118 refer to contributions from the megaplasmid pMP118.

Feature	***L. ruminis*** **ATCC 27782**	***L. ruminis*** **ATCC 25644**	***L. salivarius*** **UCC 118**
Genome size	2,066,657	2,138,893	1,827,111 (242,436)
G+C Content (%)	44.4	43.98	32.9 (32.1)
Coding genes	1901	2,251	1765 (242)
Coding density (%)	80.5	87	84.1 (75.6)
rRNA operons	6	6	7
tRNAs	67	49+	78
Pseudogenes	85	nd	49 (20)
IS elements	83	nd	32 (11)

nd: not determined, due to draft nature of genome sequence

In addition to the 1901 protein-coding regions, the genome of *L. ruminis* contains 85 predicted pseudogenes (4.3% of all coding sequences; Figure 1), characterized by the presence of in-sequence frame-shifts, deletions, stop codons, or interruption by insertion sequences (IS). A large proportion (29.4%), of the pseudogenes themselves were identified as being IS element related. Inactivation of IS elements in this manner is a common feature of bacterial genomes, and is considered a mechanism for transposition regulation [39]. The remaining 60 pseudogenes are catalogued in Additional File 1: Table 1. IS elements are a common feature of bacterial genomes. We identified eighty-three transposases (4.2% of coding sequences) representing 9 families of IS elements in the genome of *L. ruminis* ATCC 27782, with 25 characterized as pseudogenes (Additional File 2: Table 2). Seven of the nine families are present in multiple copies, with IS256, IS66, IS3, IS200/IS605 having the largest numbers of replicates, 10, 16, 19, and 25 copies respectively.

Six rRNA operons, consisting of 16S, 23S and 5S rRNA genes, were identified distributed throughout the genome. All rRNA operons were orientated in the same direction as DNA replication. Sixty seven tRNA genes, representing all 20 amino acids, were identified in the genome. Only 26 of the 67 tRNAs were located on the lagging strand, with the majority clustered at, or close to, the first of the two rRNA operons on this strand. The remaining 41 were distributed throughout the leading strand with the majority clustered around the four rRNA operons. Redundant tRNA genes were present for 18 of the 20 tRNA species, with the exceptions being those for cysteine and tryptophan.

**Figure 2:**
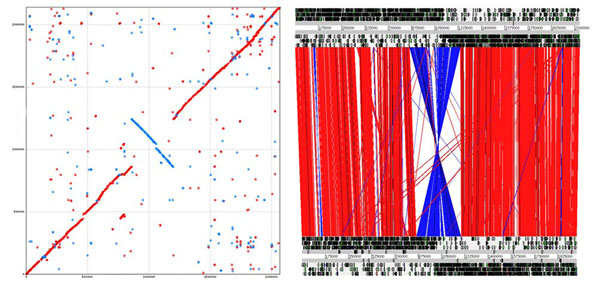
**Comparison of the genomes of two *L. ruminis* strains.** Left panel: Promer alignment of *L. ruminis* ATCC 27782 (vertical) and *L. ruminis* ATCC 25644 (horizontal) genomes. Red dots represent regions of homology between the genomes and which are in the same orientation. Blue dots represent homology between the genomes in the opposite orientation, highlighting the inversion centred around the putative replication terminus region. Right panel: ACT comparison (DNA-DNA) of *L. ruminis* ATCC 25644 (top) and *L. ruminis* ATCC 27782 (bottom).

In addition to the complete genome of *L. ruminis* ATCC 27782, we also generated a high draft-quality assembly [38] of the *L. ruminis* ATCC 25644 genome, as described in Methods. Although not assembled, projection against the ATCC 27782 genome suggests that the genome of ATCC 25644 consists of a slightly larger circular chromosome of 2,138,893 bp, with an average G+C content of 43.98%. A preliminary annotation of this draft genome identified 2,251 coding regions representing a coding density of 87%. This may be an over-estimate due to the draft quality of the genome [40]. Comparative analysis of the two *L. ruminis* genomes (Figure 2) revealed a high degree of synteny, but this is disrupted by a large chromosomal inversion centered around the replication terminus region.

*L. ruminis* is one of 12 species in the *L. salivarius* clade which have been identified as being motile (only 14 species of the genus *Lactobacillus* are known to be motile). Annotation of the *L. ruminis* ATCC 27782 genome identified all the motility and motility-associated proteins required to produce a fully functional flagellar apparatus. The genomics of *L. ruminis* motility and flagellar assembly are described in detail elsewhere [36]. To summarize, the motility-encoding regions of the ATCC25644 and ATCC27782 genomes span 45,687 bp and 48,062 bp respectively, constituting a single contiguous gene block. *L. ruminis* motility is conferred by a total of forty-five predicted proteins involved in flagellum regulation, synthesis, export and chemotaxis, and which conform to the expectations for flagellum production in Gram positive bacteria [41]. The motility locus of ATCC 27782 is larger because it includes a second copy of the gene for flagellin, *fliC*, and a glycosyltransferase pseudogene, the relevance of which for motility is unclear. The closest homolog of most of the *L. ruminis* motility genes was in *Enterococcus casseliflavus* or *Enterococcus gallinarum*, which is consistent with phylogenetic relatedness of the enterococci to the lactobacilli [42], and distribution of the motility phenotype in the phylum *Firmicutes*.

### Genomics of *L. ruminis* metabolism

The *in silico* analysis of the *L. ruminis* genome suggests that it is unable to synthesize the vitamins and cofactors riboflavin, vitamin B6, folate, nicotinamide and nicotinate. Partial pathways for both purine and pyrimidine biosynthesis were annotated (Additional File 3: Figure 1 and Additional File 4: Figure 2, respectively). However, while *L. ruminis* appears to lack the ability to synthesise adenosine and guanosine, it is predicted to synthesize the nucleotides adenine and guanine from adenosine monophospate (AMP) and guanine monophosphate (GMP) respectively.

In contrast to other *Lactobacillus* species such as *L. helveticus* and *L. sakei*, which convert pyruvate to acetyl-CoA through the intermediate acetyl phosphate, *L. ruminis* cannot produce acetyl-CoA in this manner. Instead *L. ruminis* appears to produce Acetyl-CoA through the action of the enzyme pyruvate formate-lyase (Additional File 5: Figure 3). Pyruvate formate-lyase catalyses the non-oxidative cleavage of pyruvate to acetyl-CoA and formate. An anaerobically induced pyruvate formate-lyase system has been fully characterised in *E. coli*[43].

Through *de-novo* synthesis and inter-conversions, *L. ruminis* can synthesize 8 of the 20 amino acids. Present in the genome is a gene predicted to encode the enzyme L-serine dehydratase (EC. 4.3.1.17) which catalyses the conversion of pyruvate into serine. Serine in turn can be converted by tryptophan synthase into tryptophan (Additional File 6: Figure 4). Tryptophan can also be synthesised *de novo* through the Shikimate pathway. *L. ruminis* is also predicted to be capable of d*e novo* synthesis of histidine. While the *L. ruminis* ATCC 27782 genome apparently encodes complete pathways for the production of threonine and aspartate, it lacks the enzymes threonine aldolase (EC: 4.1.2.5) and glycine hydromethyltransferase (EC: 2.1.2.1). Consequently this strain cannot synthesis glycine. *L. ruminis* is also predicted to lack the ability to synthesize glutamate. However, if extracellular glutamate is imported (two glutamate ABC transport systems are present in the genome of *L. ruminis*, LRC_13790-13800 and LRC_18670-18680), *L. ruminis* could subsequently synthesize glutamine, arginine and proline. In summary, *L. ruminis* is potentially capable of synthesizing 8 amino acids and being auxotrophic for 12. This level of auxotrophy is greater than that exhibited by its nearest sequenced neighbour *Lactobacillus salivarius* UCC118 [30] which is auxotrophic for only 8 amino acids. This highlights the dependence this autochthonous bacterium has on extracellular sources of amino acids that are likely to be present in the intestinal milieu. However, *L. ruminis* is considerably less auxotrophic than more distantly related *Lactobacillus* species such as *L. acidophilus* NCFM (auxotrophic for 14 amino acids) [44]and *L. sakei* (auxotrophic for 18 amino acids).

Apart from carbohydrate metabolism (see below), preliminary analysis of the genome of *L. ruminis* ATCC 25644 revealed a near identical predicted metabolic profile to that described for *L. ruminis* ATCC 27782. However, some subtle differences were noted; for example ATCC 25644 appears to lack the enzyme asparatate aminotransferase (EC:2.6.1.1) but possesses the enzymes 3-isopropylmalate dehydrogenase (EC:1.1.1.85), succinyl-diaminopimelate desuccinylase (EC:3.5.1.18) and aryl-alcohol dehydrogenase (EC:1.1.1.90). The two *L. ruminis* strains are predicted to be auxotrophic for the same 12 amino acids and to have identical pyruvate metabolism systems. Similar to ATCC 27782 and most other lactobacilli, *L. ruminis* ATCC 25644 cannot synthesize the majority of vitamins and co-factors.

The ability of intestinal bacteria to utilize carbohydrates is an important factor for determining competitiveness and diet interaction in the host intestine, and we describe this topic in detail elsewhere in this volume [40]. Sixteen carbohydrate utilization pathways were predicted in genomes of ATCC 27782 and ATCC 25644, including those for utilization of glucose, fructose, mannose, galactose, starch and sucrose [40]. The ATCC 25644 encodes six putative operons for the transport and utilisation of the prebiotics fructo-oligosaccharides (FOS), galacto-oligosaccharides (GOS), soya-bean oligosaccharides (SOS), and 1,3:1,4-β-D-Gluco-oligosaccharides [40]. Only three of these operons were identified in the ATCC 27782 genome, which were putatively linked to the utilisation of SOS and 1,3:1,4-β-D-Gluco-oligosaccharides. Lack of an operon for FOS utilization in the bovine isolate ATCC 27782 is consistent with the inability of this strain to use FOS as a sole carbon source. A predicted cellobiose utilization operon in the *L. ruminis* 25644 genome is likely to be responsible for the transport and hydrolysis of both cellobiose and 1,3:1,4-β-D-Glucan hydrolysates [40].

### Environment-interaction traits

Bacteriocins are small antimicrobial peptides produced by many lactic acid bacteria, that may exhibit either a narrow spectrum (affecting only closely related species) or broad spectrum (affecting species in different genera) [45]. The genome of *L. ruminis* ATCC 27782 includes a 6.1 kb region encoding seven bacteriocin-related and two hypothetical genes (Additional File 7: Figure 5). *In silico* analysis identified the bacteriocin (59 aa protein; LRC_02417) as a Class II pediocin-like bacteriocin [46]. The bacteriocin shows significant residue identity to Class II bacteriocins from *Bacillus coagulans*, *Pedicococcus acidilacti*, *L. plantarum*, and other LAB (Additional File 8: Figure 6), and possesses a conserved N terminal pediocin box region and the YGNGVXCXXXXCXV motif [47]. In addition to the bacteriocin structural gene, the locus also encodes two putative bacteriocin immunity proteins (LRC_17030 and LRC_17110), a sensor histidine kinase and response regulator (LRC_17060-17070) and transport apparatus comprising an accessory protein and ATP-binding cassette (ABC) transporter (LRC_17040 and LRC_17080). A preliminary analysis has so far failed to show bacteriocin activity associated with *L. ruminis* strain ATCC 27782, and it is not yet known if this locus is active. Analysis of the genome of ATCC 25644 also identified a region containing genes associated with bacteriocin production. However, the fragmented assembly means that it is presently unknown if the genetic complement of this locus is complete. Sequences associated with bacteriocin production were distributed across three contigs, with the genes for two sensor histidine kinases and a response regulator being truncated by sequencing gaps. Although a gene for a potential bacteriocin immunity protein (similar to PedB from *Lactobacillus gasseri*) was identified, no genes encoding bacteriocin peptides or transport apparatus were identified.

CRISPR loci (clustered regularly interspaced short palindromic repeats) are a family of DNA repeats that function like an adaptive immune response system, and are found in only 40% of bacteria. This system provides acquired immunity to exogenous DNA from viruses and plasmids [48], and thus represent a barrier to attack or genetic transformation. Two CRISPR/CRISPR-associated sequence (*cas*) systems were identified in the genome of *L. ruminis* ATCC 27782. The systems, CRISPR1 and CRISPR2, are located 12.9kb apart and consist of 8 and 7 *cas* genes respectively. CRISPR1 consists of 8 *cas* genes and is preceded by a 1059 bp CRISPR region composed of a 36bp direct repeat and 14 spacers. The CRISPR region is separated from the *cas* genes by a small hypothetical protein and a transposase fragment. CRIPSR2 consists of 7 *cas* genes and is proceeded by a much longer CRISPR region composed of a 30 bp direct repeat and 36 spacers. Analysis of both CRISPR regions revealed no significant hits to any known plasmid or phage sequences, emphasizing the phylogenetic distance of the *L. ruminis* genetic milieu from previously well characterized systems.

We identified one CRISPR system in the draft genome of *L. ruminis* ATCC 25644. CRISPR1 consists of 4 *cas* genes proceeded by a CRISPR region containing a 36 bp direct repeat (DR) and 16 spacers. The region is disrupted by a sequencing gap of 887 bp (inferred from mate-pair information) dividing the region into direct repeats with 11 and 5 spacers respectively. Given that each DR and spacer is 65 bp, the sequencing gap could contain another 13 spacers. The presence of a CRISPR system in a second *L. ruminis* genome confirms the importance of resistance to exogenous DNA in this species.

Intestinal commensal bacteria must also be able to endure a range of physiological stresses. Indeed, the ability of bacteria to respond to stresses such as those encountered during gastric and intestinal transit is key to their survival. The *L. ruminis* ATCC 27782 genome encodes a number of stress resistance proteins including those predicted to confer resistance to heat, cold, alkaline and phage shock proteins (Additional File 9: Table 3). The genome also includes the conserved SOS regulon genes. Specifically, *L. ruminis* ATCC 27782 encodes four heat shock proteins, the cold shock proteins CspA and CspE, a single alkaline shock protein, and there are two copies of *pspC* whose product is predicted to be involved in phage shock/resistance. The genome of *L. ruminis* ATCC 27782 also harbours genes for a number of Clp proteases, (*clpB*, *clpX*, and *clpP*), which are involved in the degradation of mis-folded proteins [49]

ATCC 27782 is moderately oxygen tolerant, though less so than other members of the *L. salivarius* clade [40]. Consequently, the ability of this bacterium to respond to and eliminate reactive oxygen species is extremely important. The *L. ruminis* genome encodes a number of thioredoxins, a class of protein which act as antioxidants through the reduction of other proteins by cysteine thiol-disulfide exchange [50].

### Surface proteins and carbohydrates

The *Lactobacillus* cell surface has an important role in governing interaction with host animals, at the level of initial colonization, long-term persistence, and potentially also modulatory roles on both the innate and adaptive immune responses, and the rest of the microbiota by surface exclusion [51]. Sortase enzymes function as an important mechanism which anchors surface proteins, and they are found in all Gram-positive bacteria where they act as both proteases and transpeptidases [52]. The Sortase type A enzymes (SrtA) function by anchoring proteins containing the characteristic substrate LPxTG motif to the peptidoglycan of the cell wall. Genes for two sortase-like proteins were annotated in the *L. ruminis* genome (SrtA, LRC_16570 and SrtC, LRC_00630), as well as 10 predicted sortase-anchored proteins (Additional File 10: Table 4), that were identified by searching for LPxTG motifs. The presence of multiple sortase-like proteins in the genome is not unusual in Gram-positive bacteria [53], and the NCBI protein databases currently contain 173 SrtA sequences from eight *Lactobacillus* species, plus an additional 48 SrtC sequences. The sortase-like protein encoded by LRC_00630 contains a SrtC Conserved Domain. It shows 42% BLAST identity to SrtC of *L. rhamnosus* LGG. The LRC_00630 gene is preceded by three genes predicted to encode sortase dependant proteins (LRC_00600, LRC_00610 and LRC_00620). This genetic arrangement suggest that both the genes for the sortase enzyme and its substrates may have been acquired as a unit by horizontal gene transfer, and their arrangement also suggests they may be co-transcribed or co-regulated. Both SrtA and SrtC recognize similar motifs, but the conservation of amino acids in these motifs differs i.e. LPxTGc for SrtA and lPxTGG for SrtC, where uppercase letters are absolutely conserved [52]. On this basis alone, the target proteins for the SrtA and SrtC enzymes of *L. ruminis* ATCC 27782 cannot be distinguished, and will require experimental investigation.

LRC_00600 (annotated as Sortase-anchored surface protein) is a predicted 1,140 residue protein with homology to hypothetical proteins or presumptive (but unproven) collagen adhesins. LRC_00610 (annotated as Sortase-anchored surface protein) shows 28% BLAST identity to SpaE, a minor backbone protein of the adhesive pili produced by *L. rhamnosus* LGG [54]. However, it also displays higher levels of residue identity to many putative/hypothetical sortase-dependant proteins from LAB or *Firmicutes*. LRC_00620 (505 amino acid residues) shows significant residue identity to homologues primarily in the *Enterococcus* spp,*.* including pilin subunits from *E. faecalis* and *E. faecium*. It is therefore possible that this locus encodes a sortase-dependent pilus organelle. Genetic evidence for possible production of such structures has been noted in *L. johnsonii*[55] and other lactobacilli [51], but their visualization and characterization has only been described for *L. rhamnosus* LGG (as noted above). When transcription of the LRC_00600-00630 locus in ATCC 27782 and ATCC 25644 was examined by microarray analysis, we observed that these genes were significantly up-regulated in the human isolate ATCC 25644 compared to the bovine isolate ATCC 27782, by factors of 15.2, 14.3, 7.1 and 23.8 respectively. While highly suggestive of a surface role in this strain, these presumptive pili are not visible under the conditions routinely used for negative staining (see below), and direct experimental verification by another method is now required.

There is no clustering of genes for sortase dependant proteins around the gene for the second sortase-like enzyme (LRC_16570) which we annotated as SrtA. The genes for the remaining sortase-dependant proteins are distributed throughout the genome, with another three-gene cluster in (LRC_16760, LRC_16780, LRC_16790) in the latter half of the genome. The biological function of these proteins is not known (Additional File 10), and their characterization will require a functional genomics approach as deployed for the closely related *L salivarius*[56], and *L. acidophilus*[57].

**Figure 3:**
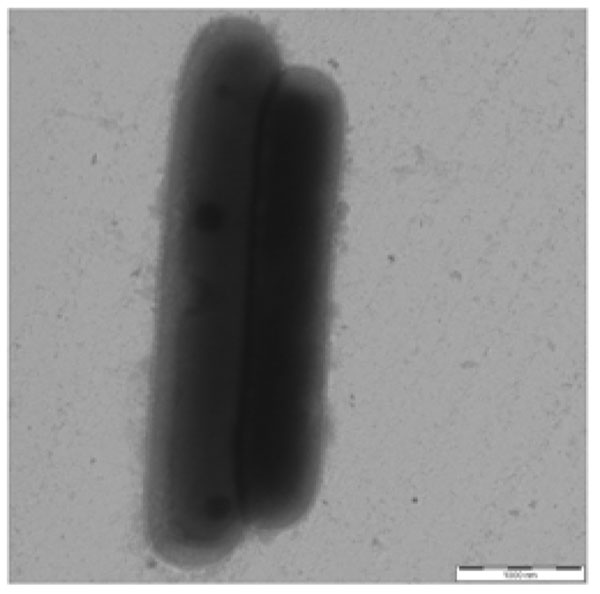
**Transmission electron microscopy of *Lactobacillus ruminis* ATCC 25644.** Cell were stained with 0.25% ammonium molybdate; 20,000 x magnification. Scale bar: 1 μm.

In contrast to the *L. salivarius* genome which harbours two major gene clusters for exopolysaccharide (EPS) production [30,58], the genome of *L. ruminis* ATCC 27782 contains only one EPS cluster, similar to the genomes of *L. acidophilus*[44], *L. johnsonii*[*21*] and *L. rhamnosus*[59]. The *L. ruminis* ATCC 27782 EPS gene cluster spans 69,912 bp (3.4% of total genome), and incorporates 62 predicted coding sequences (Additional File 11: Figure 7). The cluster contains genes for a single predicted chain length determinator, an oligosaccharide translocase, a flippase, 9 glycosyltransferases, and a priming glucose phosphotransferase (LRC_01410; Additional File 11: Figure 7). The EPS cluster also contains 16 hypothetical proteins, 6 of which are hypothetical membrane proteins, and four IS element-related proteins (transposases). The *L. ruminis* EPS gene clusters exhibits an atypical G+C content relative to the rest of the genome; the G+C content of the EPS locus is 39.66%, compared to 44.4% for the genome. It is also interesting to note that many of the genes in the EPS cluster do not have their closest homologue amongst the Lactobacilli, but instead have their closest homologues in other genera such as *Ruminococcus*, *Eubacteria* and *Butyrovibrio* (see Additional File 12: Table 5). This suggests that acquisition of the *L. ruminis* EPS-encoding region was by horizontal gene transfer in the intestinal environment, and it is tempting to theorise that some particular selective pressure was required to promote acquisition from outside the genus. Analysis of cells of *L. ruminis* by transmission electron microscopy did not clearly identify the presence of an EPS layer (Figure 3). However, it is known that EPS production in lactobacilli including the closely related *L. salivarius* species is heavily dependent on culture factors especially carbohydrate in the medium [58], variations of which were not tested in this preliminary analysis.

In addition to sortase anchored proteins the *L. ruminis* ATCC 27782 genome also encodes a predicted fibronectin binding protein (LRC_09530) and a number of proteins expected to be involved in the export and synthesis of teichoic acids (LRC_01020, LRC_01380, LRC_03490, LRC_17520, LRC_06890, LRC_06900). Additionally, the ATCC27782 genome includes the *dlt* operon (*dltA* to *dltD*; LRC_17120 to LRC_17150) involved in the esterification of lipoteichoic acid (LTA) by D-alanine, which suggests the presence of lipoteichoic acids in the *L. ruminis* cell wall.

### Comparative genomics of *L. ruminis*

Since this study provided the first complete genome sequence information for a member of the *L. salivarius* clade other than *L. salivarius* itself, we initially compared the *L. ruminis* ATCC 27782 genome to that of *L. salivarius* UCC118. *L. ruminis* is robustly positioned in the *L. salivarius* clade by independent analyses [5,42]. At summary statistic level (Table 1), the genomes of *L. ruminis* and *L. salivarius* are very similar, reflecting the close phylogenetic relationship of these two species. However, one major difference is the abundance of extra-chromosomal elements in *L. salivarius*. While *L. ruminis* has a single circular genome of 2.06 Mb, the *L. salivarius* UCC118 genome comprises a 1.8 Mb chromosome and possesses 3 plasmids, one of which is 242kb in size [30]. Multiple plasmids including megaplasmids are present in all *L. salivarius* strains tested to date [60]. Notwithstanding this difference in architecture, the genomes of *L. ruminis* and *L. salivarius* share a similar number of coding sequences, rRNA operons and tRNA genes (Table 1). Notably, the *L. ruminis* ATCC 27782 genome harbours a larger number of pseudogenes (85 compared to 69) and more IS elements (83 compared to 43). The greater number of pseudogenes and smaller genome size may indicate that the *L. ruminis* genome is at a more advanced stage of decay than *L. salivarius*, relative to their last common ancestor which was presumably free-living and had a larger genome.

**Figure 4:**
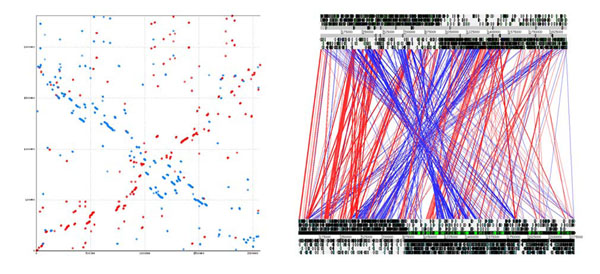
**Comparison of the genomes of *L. ruminis* and *L. salivarius*.** Left panel: Promer plot (amino acid level) comparison of the genomes of *L. ruminis* ATCC 27782 (horizontal axis) and *L. salivarius* UCC118 (vertical axis). Right panel: ACT comparison (DNA-DNA) of the genomes of *L. ruminis* ATCC 27782 (top) and *L. salivarius* UCC 118 (bottom)

**Figure 5:**
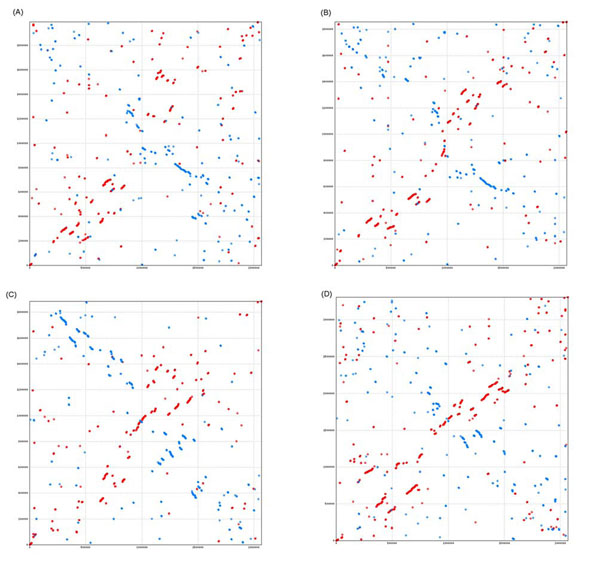
**Comparison of the genomes of *L. ruminis* with those of selected lactobacilli outside the *L. salivarius* clade.** Promer plots (amino acid level) comparisons of the genome of *L. ruminis* ATCC 27782 (horizontal axis) with the genomes (vertical axes) of (A) *L. acidophilus* (B) *L. delbrueckii* (C) *L. sakei* (D) *L. plantarum*.

In contrast to their similarity at a general category level, there is an absence of synteny between the genomes of *L. ruminis* and *L. salivarius* (Figure 4). In the Promer comparison, the genome backbone is just apparent as a diagonal of in-register orthology. The X-shaped pattern characteristic of recombination around the replication origin-terminus axis, that we previously described in phylogenetically more distant *Lactobacillus* comparisons [42], is also evident. In the ACT comparison, it is clear that large-scale re-arrangement and inversion has almost eliminated the vestiges of synteny, recalling that these two genomes are nonetheless derived from members of one of the more cohesive *Lactobacillus* clades. Thus, the extreme diversity of the genus *Lactobacillus* is manifest in the large number of member species and establishment of multiple divisions [6,9], and is replicated even within the phylogenetic clades, where the most closely related species demonstrate an unusually high level of diversity. When we compared the *L. ruminis* genome to four other species (Figure 5), there was also a lack of long-range synteny, even less than that the little observed between *L. salivarius* and *L. ruminis*.

To further examine this phenomenon, we investigated core proteins which we determined using METAPHORE [61] (see Methods), first within the *L. salivarius* clade (*L. salivarius* and *L. ruminis* genomes). A protein was considered an ortholog if it shared 30% amino acid identity over 80% of the sequence length. Only 59% of the protein coding regions (ie excluding IS elements and pseudogenes) in the *L. ruminis* genome have an ortholog in the *L. salivarius* UCC 118 genome. Including the *L. salivarius* megaplasmid in the analysis, the genomes of *L. ruminis* and *L. salivarius* contained 309 and 358 genes, respectively, which were absent in the other genome at the cut-off value for orthology imposed for their proteins (Additional File 13: Table 6 for *L. ruminis*–specific proteins, and Additional File 14: Table 7 for *L. salivarius*-specific proteins). However, a large proportion of these unique proteins in each genome corresponded to hypothetical genes (97 in *L. ruminis* and 115 in *L. salivarius*). A further 58 unique *L. salivarius* proteins were associated with prophages compared to only 11 in the *L. ruminis* genome. The *L. ruminis* SrtC homolog (LRC_00630) and two of its sortase dependant proteins (LRC_00600, LRC_00610) are absent from the *L. salivarius* genome, as are 9 of the CRISPR associated proteins. The presence of only 1 small CRISPR region in the genome of *L. salivarius* may account for the greater abundance of phage associated genes within its genome. The *L. ruminis-*specific proteins include those for motility [36], ability to utilize certain carbohydrates such as cellobiose [40], and a large number of predicted membrane proteins of unknown function (Additional File 13: Table 6). The previously discussed pediocin-like bacteriocin was also identified by this analysis. The complement of *L. salivarius*-specific proteins is striking for how many of them are encoded by discrete tracts of the genome, even outside of phage-related sequences, exemplified by LSL_0330 to LSL_0365 and LSL_0410 to LSL_0476 (many predicted membrane proteins); LSL_0921 to LSL_0963 (a cluster of hypothetical proteins); and the two EPS clusters [58]. Some of these regions are also evident from the ACT comparison (Figure 4), as discrete regions where homology is lacking between the genomes. This suggests that regions were differentially retained from the last common ancestor of the *L. salivarius* clade – or differentially acquired. The average GC% of unique genes for the genomes of *L. ruminis* ATCC 27782 and *L. salivarius* UCC118 was 42.7% and 31.9% respectively. However the GC% ranges were from 26.2% to 57.3% for *L. ruminis* and from 21.5% to 45% for *L. salivarius*, indicating that a number of genes unique to each genome may have been acquired by horizontal gene transfer.

Due to the lack of any other sequenced species from this subgroup, the 1,100 proteins conserved in both genomes were considered the core proteins of the *L. salivarius* clade. The majority of the core proteins have a defined function with only 166 hypothetical proteins (35% of the total number of hypothetical proteins) and 189 hypothetical proteins (32 % of the total number of hypothetical proteins) in *L. ruminis* and *L. salivarius* respectively. More comprehensive manual comparative analysis (data not shown) revealed that the core protein set of the *L. salivarius* clade was predominated by genes present in operon-like clusters, an organization which has previously been noted in another study of core genes in the Lactobacilli [62], suggesting conserved function, organization and control of such core genes. In addition to housekeeping genes and clusters of ribosomal and ATPase proteins, *L. ruminis* and *L. salivarius* share a clusters of genes involved in EPS production and purine metabolism. Five two-component regulatory systems were shared between both genomes and while their function is currently unknown, they may form the basis of environmental response systems shared by members of this clade.

To determine relatedness levels with a broader sampling of the genus, we compared the core proteins of the *L. salivarius* clade with those in five other groups of lactobacilli. These were based upon representative sampling of major groups defined in our previous phylogenetic analyses [42] as follows: Group A, *L. acidophilus* and *L. johnsonii*; Group B, *L. reuteri* and *L. fermentum*; Group C, *L. brevis* and *L. buchneri*; Group D, *L. plantarum* only (*L. plantarum* is the only sequenced member of this group); and Group X (not defined as a specific group in Canchaya et al, 2006), *L. casei* and *L. sakei*. We first defined the core proteins in each group using METAPHORE ([61]; see Methods). Table 2 shows that the number of orthologous proteins for each species-pair in a Group was reasonably constant, ignoring Group D. The number of core proteins shared by a particular group and the *L. salivarius* clade core protein set was proportional to the 16S rRNA gene phylogenetic distance. This is as would be expected from our previous usage of this number for phylogenomic comparison [42]. The number of unique proteins in each Group (relative to the *L. salivarius* clade core protein set) was less closely correlated with phylogenetic distance from *L. salivarius*–*L. ruminis*.

**Table 2:** Comparative analysis of orthologues shared between the *L. salivarius* clade and selected lactobacillus groups.

**Group**	**Members analyzed**	**Orthologs^a^**	**Core proteins^b^**	**Unique proteins^c^**
A	*L. acidophilus*, *L. johnsonii*;	1277	760	242 (168)
B	*L. reuteri*, *L. fermentum*	1216	810	189 (135)
C	*L. brevis*, *L. buchneri*	1382	830	241 (145)
D	*L. plantarum*	3009	975	840 (68)
X	*L. casei*, *L. sakei*	1214	822	178 (143)
				

a. The number of orthologs shared between the two members of the indicated lactobacillus group.

b. The number of orthologs shared between the core set of the *L. salivarius* clade and the indicated lactobacillus group

c. The number of proteins in the indicated lactobacillus groups which are not present in the L. salivarius clade core protein set. Numbers in brackets represent the number of proteins in the core protein set of the *L. salivarius* clade which are absent in the indicated Lb group.

We also identified 517 proteins that were common to all six *Lactobacillus* groups (Additional File 15; Table 8), where the sixth group, Group E, is the *L. salivarius* clade, for consistency with Canchaya et al, 2006 [42]). In addition to the expected housekeeping proteins, ribosomal proteins and ATPase proteins, the 6 groups share three two-component regulatory systems which may form the basis of environmental response systems shared by all analyzed members of the genus (Additional File 15; Table 8). Additionally, 41 hypothetical proteins, including 4 hypothetical membrane proteins, appear to be conserved across the six groups. Table 3 shows the numbers of unique proteins that were present in a given lactobacillus group but absent in the combined lactobacillus core protein set from all the other groups – in other words, group-unique core proteins. Group D contained the largest number of unique proteins, reflecting the larger genome of *L. plantarum* (Table 3). No group appears to possess any unique proteins associated with niche adaption or environment-interaction (see Additional File 16; Table 9 for protein identities by group).

**Table 3:** Unique proteins in selected lactobacillus groups.

**Group**	**Members analyzed**	**Unique proteins**
A	*L. acidophilus*, *L. johnsonii*;	35
B	*L. reuteri*, *L. fermentum*	6
C	*L. brevis*, *L. buchneri*	9
D	*L. plantarum*	77
E	*L. salivarius* , *L. ruminis*	9
X	*L. casei*, *L. sakei*	10

[63][61]

## Conclusions

The genome sequences of these two *L. ruminis* strains provide a platform for functional genomic analysis of this species, an overlooked autochthonous member of the intestinal microbiota of many animals including humans. Similar to other commensal lactobacilli, the *in silico* analysis of the *L. ruminis* genome suggested it may be undergoing genome decay. The comparative analysis of *L. ruminis* ATCC 27782 and *L. salivarius* UCC118 revealed a lack of genome synteny between these two members of the *L. salivarius* clade which reflects the high degree of diversity evident across the whole genus. Adaptations to a competitive environment in the intestine include a large locus devoted to EPS production by *L. ruminis*, a pediocin-like bacteriocin locus, and a putative sortase-dependent pilus locus that is expressed at higher levels in the strain isolated from humans.

## Methods

## Genome sequencing and annotation

The genomes of both *L. ruminis* ATCC 25644 and *L. ruminis* ATCC 27782 were sequenced by generating approximately 200,000 reads of average read length 125-150 nt, from a half plate on a 454 FLX instrument [64], using a 3 kb mate pair library, generating approximately 21-fold and 28-fold coverage (Agincourt Biosciences, Beverly, MA), respectively. In addition to the 454 data for the ATCC 27782 genome, an additional half lane of Illumina sequencing (22.5 Mb total sequence data) was obtained. The Illumina data consisted of a 3 kb mate-pair library and a 400 bp paired-end library (Fasteris, Geneva, Switzerland). Each Illumina library provided an average of 217-fold coverage. Initial *de novo* genome assembly of the 454 sequences was performed using the Roche/454 Life Sciences Newbler (Gs) assembler [65], producing an initial assembly of 72 contigs distributed over 8 scaffolds for the genome of ATCC 27782. The resulting 454 assembly was then used as a reference for the mapping assembly of the Illumina data. This mapping assembly was performed using Mira [66]and undertaken to extend contigs, close gaps and for error correction of the draft genome.

A PCR-based strategy was adopted for gap closure. Contig-contig gaps were closed using primers designed at the end of contigs and amplified using Dreamtaq DNA polymerase (Fermentas, Ontario, Canada). Scaffolds were ordered and oriented by PCR. Primers were designed at the ends of the scaffolds and the inter-scaffold region was amplified using Extensor long PCR enzyme mix (Abgene, Epsom, UK). PCR products for both the sequencing gaps and the inter-scaffold gaps were sequenced by Eurofins MWG Operon (Ebersberg, Germany) and the sequences were intergrated into the assembly using PHRAP [67]. Correct placement of the gap sequences was confirmed by observation using Tablet, a next generation sequencing graphical viewer [68].

Initial automated gene calling was performed using Glimmer 3 [69] and Genemark [70]. Intergenic regions were examined for missed gene calls using BlastXtract [71]. tRNAs were identified using tRNA-scan [72] and ribosomal binding sites using RBSfinder [73]. Preceding the manual annotation of the *L. ruminis* ATCC 27782 genome, the protein sequences of each gene product were searched against a variety of databases with the aim of assigning a functional annotation. All predicted proteins were searched (BLASTP) against the NCBI-non-redundant protein database (nr) and, through Interproscan [74], against the pFAM, TigrFAM, PIR, HAMAP, PROSITE, PRINTS, PRODOM, PANTHER, SUPERFAMILY, GENE3D databases. In addition, transmembrane domains were identified with TMHMM [75] and Signal peptides with SignalP [76]. The automated annotation was then manually curated in Artemis [77].

Accession numbers: The finished genome of ATCC 27782 is available under accession number XXYYZZ123. The draft genome of ATCC 25644 is available under accession number CCGGHHIIUU.

### Genome comparisons

Whole genome nucleotide alignments were generated using the Big Blast software (available from the Welcome Trust Sanger Institute [78] and alignments were visualized with the Artemis Comparison Tool (ACT) [79]. Protein alignments were performed using the MUMmer package [80]. Identification of orthologs, unique genes and core genes was performed using the custom in-house software METAPHORE [61]. METAPHORE performs a bi-directional blastp comparison of two or more genomes and proteins are only considered orthologs if they share a minimium 30% amino acid identity over 80% of their sequence length. For an ortholog to be considered a core gene, it must be present in all possible pairwise genome combinations.

### Transcriptome analysis

Microarray production, scanning and data analysis followed an established protocol [79]. In summary, *L. ruminis* cells were grown anaerobically for 15 hrs in 20 ml de Man-Rogosa-Sharpe (MRS) broth aliquots until the OD_600_ was in the range of 0.5-0.8. The cells were harvested by centrifugation at room temperature and the pellets were immediately washed and resuspended in 500 µl RNAprotect Bacteria Reagent (Qiagen). Total RNA was extracted using an RNeasy mini kit (Qiagen), according to the manufacturer’s protocol for difficult to lyse cells with modifications including an extended incubation with proteinase K (40 mins). RNA was treated with DNase using the Turbo DNA-free kit (Ambion) according to the routine DNase treatment protocol. Then, 10 ug of total RNA was reverse transcribed with random nonomers (MWG-Biotech, Germany) and the ULS cDNA synthesis and labelling kit (Kreatech, Amsterdam, Netherlands). Labelling took place at 85°C for one hour.

Custom oligonucleotide microarrays that were designed to include the annotated open reading frames of the *L. ruminis* ATCC 25644 and ATCC 27782 genomes were commissioned and produced by Agilent Ltd. (Santa Clara, California). Four 44 K microarrays were present on each slide. Every 1000 nt of coding sequence was represented on the arrays by at least six features. Where the sequence of a given probe was identical for a gene common to ATCC 25644 and ATCC 27782, the probe was represented on the array six, rather than twelve times. A total of fourteen user defined control probes were represented ten times on each array in addition to the 1417 Agilent controls.

An Oligo aCGH/ChIP-on chip hybridization kit (Agilent) was used for hybridisation of the labelled cDNA to the microarrays. Probe hybridization took place at 65°C for 20 hrs with constant rotation (10 rpm). Microarrays were scanned using the Agilent Microarray Scanner System (G2505B) and the scanned files were converted to data files with Feature Extraction software (Aglient, version 9.1). Outliers were identified and removed using the Grubbs test [81] and the mean of replicate probes was calculated. The Cyber-T test [82] was employed to calculate p-values. Significance was apportioned to genes with an expression ratio ≥5 and a p-value of ≤1.0x10^-4^. Final expression ratios presented are the average of three biological replicates.

### List of abbreviations used

aa: amino acid; ACT: Artemis comparison tool; AMP: adenosine monophosphate; BLAST: Basic Local Alignment Search Tool; Bp: Base pairs; CRISR: Clustered Regularly Interspaced Short Palindromic Repeats; CAS: CRISPR-associated sequence; DR: direct repeat; EPS: Exopolysaccharide; GIT: Gastrointestinal tract; GMP: guanine monophosphate; IS: insertion sequence; LAB: Lactic Acid Bacteria; NCBI: National Center for Biotechnology Information; NF- κB: nuclear factor; PCR: polymerase chain reaction; nr: Nonredundant protein database; Nt: Nucleotides; TNF: tumour necrosis factor;

## Competing interests

The authors declare they have no competing interest.

## Authors' contributions

BMF and BAN performed research, analyzed data and drafted the manuscript; MMOD, ERB and MJC analyzed data; RPR co-conceived the research and revised the manuscript; AC performed research and analyzed data; PWOT co-conceived the research, analyzed data and drafted the manuscript.

## Supplementary Material

Additional File 1 Pseudogenes identified in the *L. ruminis* ATCC 27782 genome. Click here for file

Additional File 2 IS elements identified in the *L. ruminis* ATCC 27782 genome Click here for file

Additional File 3 Purine metabolism of *L. ruminis* ATCC 27782. Enzyme labels in green boxes represent those for which the corresponding gene was annotated in the genome. Click here for file

Additional File 4 Pyrimidine metabolism of *L. ruminis* ATCC 27782. Enzyme labels in green boxes represent those for which the corresponding gene was annotated in the genome. Click here for file

Additional File 5 Pyruvate metabolism of *L. ruminis* ATCC 27782. Enzyme labels in green boxes represent those for which the corresponding gene was annotated in the genome. Click here for file

Additional File 6 Partial metabolic map of *L. ruminis* ATCC 27782, showing the predicted inter-conversions of pyruvate, serine, and tryptophan. Enzyme labels in green boxes represent those for which the corresponding gene was annotated in the genome. Click here for file

Additional File 7 Schematic diagram of the locus encoding a putative Class IIa bacteriocin locus of *L. ruminis* ATCC 27782. Numbers above the diagram are nucleotide co-ordinates in the genome. Labels below the line are locus tags. Click here for file

Additional File 8 Multiple sequence alignment of the putative bacteriocin encoded by the LRC_17050 gene of *L. ruminis* ATCC 27782, and other Class II bacteriocin proteins, modified from Nissen-Meyer 2009, and Rea 2011 [46, 83]. Residues are numbered, by convention, with residue 1 being the first residue before the YGNG motif [46]. Click here for file

Additional file 9 *L. ruminis* stress resistance proteins Click here for file

Additional File 10 *L. ruminis* sortase enzymes and sortase anchored proteins Click here for file

Additional File 11 Schematic diagram of a gene cluster predicted to encode EPS biosynthesis genes Click here for file

Additional File 12 Annotation and phylogenetic relatedness of the EPS production locus of *L. ruminis* ATCC27782. Click here for file

Additional File 13 *L. ruminis*–specific proteins as determined by comparison with *L. salivarius* *Click here for file*

Additional File 14 *L. salivarius*-specific proteins as determined by comparison with *L. ruminis* *Click here for file*

Additional File 15 Proteins that were common to all six *Lactobacillus* groups analyzed Click here for file

Additional File 16 Proteins unique to six lactobacillus groups relative to the combined protein set of all other species in the analysis Click here for file
