# Supplementary material for: Genome sequences and comparative genomics of two Lactobacillus ruminis strains from the bovine and human intestinal tracts
Source: Microb Cell Fact. 2011 Aug 30;10(Suppl 1):S13. doi: 10.1186/1475-2859-10-S1-S13 (PMC3231920; doi:10.1186/1475-2859-10-S1-S13)
Supplement: Additional File 1 — Pseudogenes identified in the L. ruminis ATCC 27782 genome. [file 1475-2859-10-S1-S13-S1.pdf]

| Locus tag | Start | Stop | Product |
|---|---|---|---|
| LRC_01100 | 124720 | 125100 | Hypothetical protein |
| LRC_01210 | 135617 | 139665 | Bacterial membrane protein |
| LRC_01250 | 142785 | 144298 | yfhO membrane protein |
| LRC_01940 | 216308 | 216938 | ABC transporter, ATP-binding protein |
| LRC_02220 | 243885 | 244046 | Acetylxylan esterase |
| LRC_02330 | 254635 | 254892 | Hypothetical protein fragment |
| LRC_02340 | 254838 | 255071 | Hypothetical protein fragment |
| LRC_02350 | 255095 | 255280 | Hypothetical protein fragment |
| LRC_02450 | 263230 | 263481 | Hypothetical protein |
| LRC_03690 | 390206 | 390583 | Type II restriction endonuclease |
| LRC_03730 | 392695 | 393387 | Glutamine amidotransferases |
| LRC_04370 | 476288 | 476623 | Geranyltranstransferase |
| LRC_04680 | 508520 | 509487 | Biotin operon repressor |
| LRC_04700 | 509826 | 510185 | Resolvase |
| LRC_04790 | 517944 | 519543 | CTP synthetase |
| LRC_04850 | 525022 | 527534 | Heavy-metal transporting P-type ATPase |
| LRC_04880 | 529145 | 529510 | Transcriptional regulator AraC family |
| LRC_05190 | 565368 | 567844 | Recombinase |
| LRC_05210 | 568590 | 570711 | DNA (cytosine-5-)-methyltransferase |
| LRC_05360 | 583252 | 585183 | Restriction enzyme BgcI subunit alpha |
| LRC_05390 | 587516 | 587662 | Restriction enzyme fragment |
| LRC_05990 | 644266 | 644857 | Acetyltransferase |
| LRC_06340 | 683795 | 684728 | Conserved hypothetical protein |
| LRC_06890 | 739403 | 739885 | GtcA family membrane protein |
| LRC_07450 | 797582 | 798319 | Phage-related membrane protein |
| LRC_07720 | 825724 | 827078 | Conserved hypothetical protein |
| LRC_09630 | 1011987 | 1012837 | nicotinate-nucleotide pyrophosphorylase |
| LRC_09750 | 1023762 | 1024285 | dihydrofolate reductase |
| LRC_10180 | 1065304 | 1066166 | conserved hypothetical protein |
| LRC_10290 | 1078925 | 1079305 | Glucanotransferase fragment |
| LRC_10310 | 1080807 | 1081004 | Hypothetical protein |
| LRC_10320 | 1081085 | 1081732 | Conserved hypothetical protein |
| LRC_10560 | 1110091 | 1112410 | Single-stranded-DNA-specific exonuclease |
| LRC_10900 | 1140324 | 1140662 | Polysaccharide deacetylase |
| LRC_11070 | 1153189 | 1153680 | ABC transporter-related protein |
| LRC_11090 | 1154846 | 1154980 | DeoR family transcriptional regulator |
| LRC_11100 | 1155155 | 1155337 | Hypothetical protein |
| LRC_11180 | 1164422 | 1166541 | Multimodular transpeptidase-transglycosylase PBP 2A |
| LRC_11200 | 1167281 | 1168271 | ABC-type oligopeptide transport system, periplasmic component fragment |
| LRC_13200 | 1372082 | 1376966 | Restriction-modification system LlaBIII |
| LRC_13390 | 1400957 | 1403367 | Conserved hypothetical protein |
| LRC_14560 | 1515931 | 1516533 | Phosphoesterase |
| LRC_14580 | 1518657 | 1520031 | Amino acid permease |
| LRC_15260 | 1575358 | 1577797 | Glycogen phosphorylase |
| LRC_15290 | 1580399 | 1581542 | glucose-1-phosphate adenyltransferase |
| LRC_16690 | 1728915 | 1729545 | amino acid transport protein |
| LRC_17220 | 1775683 | 1776802 | Histidinol-phosphate transaminase |
| LRC_17380 | 1791692 | 1792340 | ABC transporter, ATP-binding protein |
| LRC_18240 | 1873276 | 1875472 | Alpha-galactosidase |
| LRC_18570 | 1909107 | 1909337 | DNA-binding protein |
| LRC_18630 | 1913905 | 1914436 | Signal peptidase I |
| LRC_18660 | 1917772 | 1919194 | Cell wall-associated hydrolase |
| LRC_18830 | 1943093 | 1943714 | 6-phospho-beta-glucosidase |
| LRC_18990 | 1967565 | 1967843 | Chorismate mutase |
| LRC_19020 | 1970735 | 1970995 | HAD superfamily hydrolase |
| LRC_19420 | 2019270 | 2020413 | Conserved hypothetical membrane protein |

| | | | |
|---|---|---|---|
| LRC_19460 | 2024632 | 2025139 | Isochorismatase/hydrolase |
| LRC_19480 | 2025941 | 2026090 | Phosphate ABC superfamily ATP binding cassette transporter, binding protein |
| LRC_19510 | 2026550 | 2027901 | MATE efflux family protein |
| LRC_19800 | 2059174 | 2064388 | Cell wall-associated serine proteinase |
