# Supplementary material for: Genome sequences and comparative genomics of two Lactobacillus ruminis strains from the bovine and human intestinal tracts
Source: Microb Cell Fact. 2011 Aug 30;10(Suppl 1):S13. doi: 10.1186/1475-2859-10-S1-S13 (PMC3231920; doi:10.1186/1475-2859-10-S1-S13)
Supplement: Additional File 2 — IS elements identified in the L. ruminis ATCC 27782 genome [file 1475-2859-10-S1-S13-S2.pdf]

Locus tag	Start	Stop	IS Family
LRC_00500	55965	56309	IS200/IS605
LRC_00510	56306	57491	IS200/IS605
LRC_01050	119423	119749	IS66
LRC_01080	122273	122587	IS3
LRC_01220	136245	137483	IS256
LRC_01950	217128	218363	IS200/IS605
LRC_02020	224680	225213	IS3
LRC_02030	225210	226112	IS3
LRC_02270	248735	248902	IS200/IS605
LRC_02440	261810	263050	IS256
LRC_02460	263571	264473	IS3
LRC_02470	264470	265003	IS3
LRC_02670	297367	297699	IS66
LRC_02680	297693	299288	IS66
LRC_03440	365165	366364	IS21
LRC_03580	380903	382031	IS256
LRC_04310	471329	471664	IS3
LRC_04320	471708	472166	IS3
LRC_04330	472296	473140	IS4
LRC_04350	474636	475223	IS4
LRC_04380	476748	477281	IS3
LRC_04600	500360	501898	Is66
LRC_04630	502639	503607	IS256
LRC_04690	509587	509766	IS21
LRC_04860	527733	528831	IS200/IS605
LRC_05010	543281	544519	IS256
LRC_05760	624442	624849	IS3
LRC_05770	624965	625867	IS3
LRC_06360	687723	688850	IS256
LRC_06720	721361	722040	ISL3
LRC_06840	733259	734327	IS607
LRC_07050	765607	765849	IS66
LRC_07060	766022	767526	IS66
LRC_07470	798943	799201	IS66
LRC_08020	855420	856547	IS256
LRC_08060	859422	860156	IS3
LRC_08250	878213	878446	IS200/IS605
LRC_08260	878533	879639	IS200/IS605
LRC_08430	894212	894688	IS3
LRC_08440	894685	895587	IS3
LRC_08560	903963	905090	IS256
LRC_08860	939609	939941	IS200/IS605
LRC_08870	939949	941130	IS200/IS605
LRC_10000	1046523	1046687	IS66
LRC_10140	1062588	1064147	IS66
LRC_10150	1064266	1064508	IS66
LRC_10300	1079428	1080672	IS200/IS605
LRC_10370	1087291	1088187	IS3
LRC_10380	1088190	1088723	IS3
LRC_10690	1122722	1122817	IS200/IS605
LRC_11060	1153047	1153169	na
LRC_11440	1197049	1197762	IS3
LRC_12270	1276995	1277318	IS200/IS605
LRC_12280	1277827	1278933	IS200/IS605
LRC_12820	1335459	1335623	IS200/IS605
LRC_13400	1401921	1403048	IS256
LRC_13410	1403433	1403870	IS200/IS605
LRC_13420	1404217	1404333	IS200/IS605

LRC_13480	1410478	1411974	IS66
LRC_13490	1412067	1412399	IS66
LRC_13840	1440941	1441879	IS200/IS605
LRC_13850	1441947	1442774	IS200/IS605
LRC_13860	1443268	1444071	ISL3
LRC_14630	1522457	1523626	IS200/IS605
LRC_14640	1523623	1524057	IS200/IS605
LRC_15000	1555433	1556146	IS3
LRC_15340	1586460	1588055	IS66
LRC_15350	1588049	1588402	IS66
LRC_15480	1603337	1603624	IS21
LRC_15490	1604075	1605908	IS21
LRC_15500	1604685	1605908	IS21
LRC_15520	1607765	1609078	IS30
LRC_16650	1722921	1723253	IS66
LRC_16660	1723283	1724842	IS66
LRC_16970	1752785	1754023	IS256
LRC_17530	1810531	1810785	IS200/IS605
LRC_18120	1864086	1864415	IS200/IS605
LRC_18130	1864417	1865268	IS200/IS605
LRC_18140	1865265	1865585	IS200/IS605
LRC_19380	2014117	2014831	IS3
LRC_19520	2028301	2029494	IS200/IS605
LRC_19530	2029494	2029904	IS200/IS605
LRC_19550	2030751	2031277	ISL3
LRC_19780	2057520	2058053	IS3
