# Supplementary figures and images for: Genome sequences and comparative genomics of two Lactobacillus ruminis strains from the bovine and human intestinal tracts
Source: Microb Cell Fact. 2011 Aug 30;10(Suppl 1):S13. doi: 10.1186/1475-2859-10-S1-S13 (PMC3231920; doi:10.1186/1475-2859-10-S1-S13)

# PURINE METABOLISM

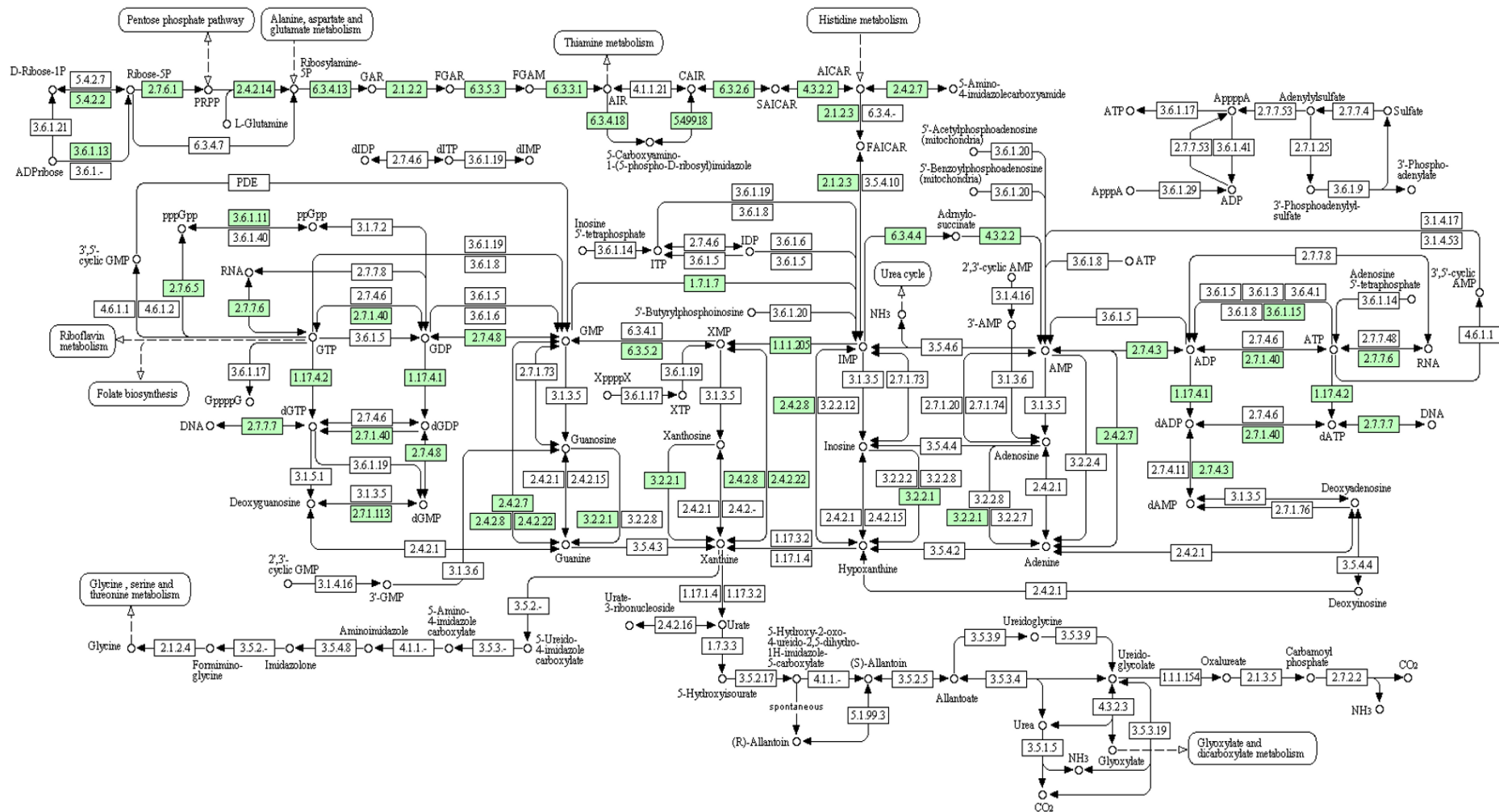

Supplement: Additional File 3 — Purine metabolism of L. ruminis ATCC 27782. Enzyme labels in green boxes represent those for which the corresponding gene was annotated in the genome. [file 1475-2859-10-S1-S13-S3.pdf]

# PYRIMIDINE METABOLISM

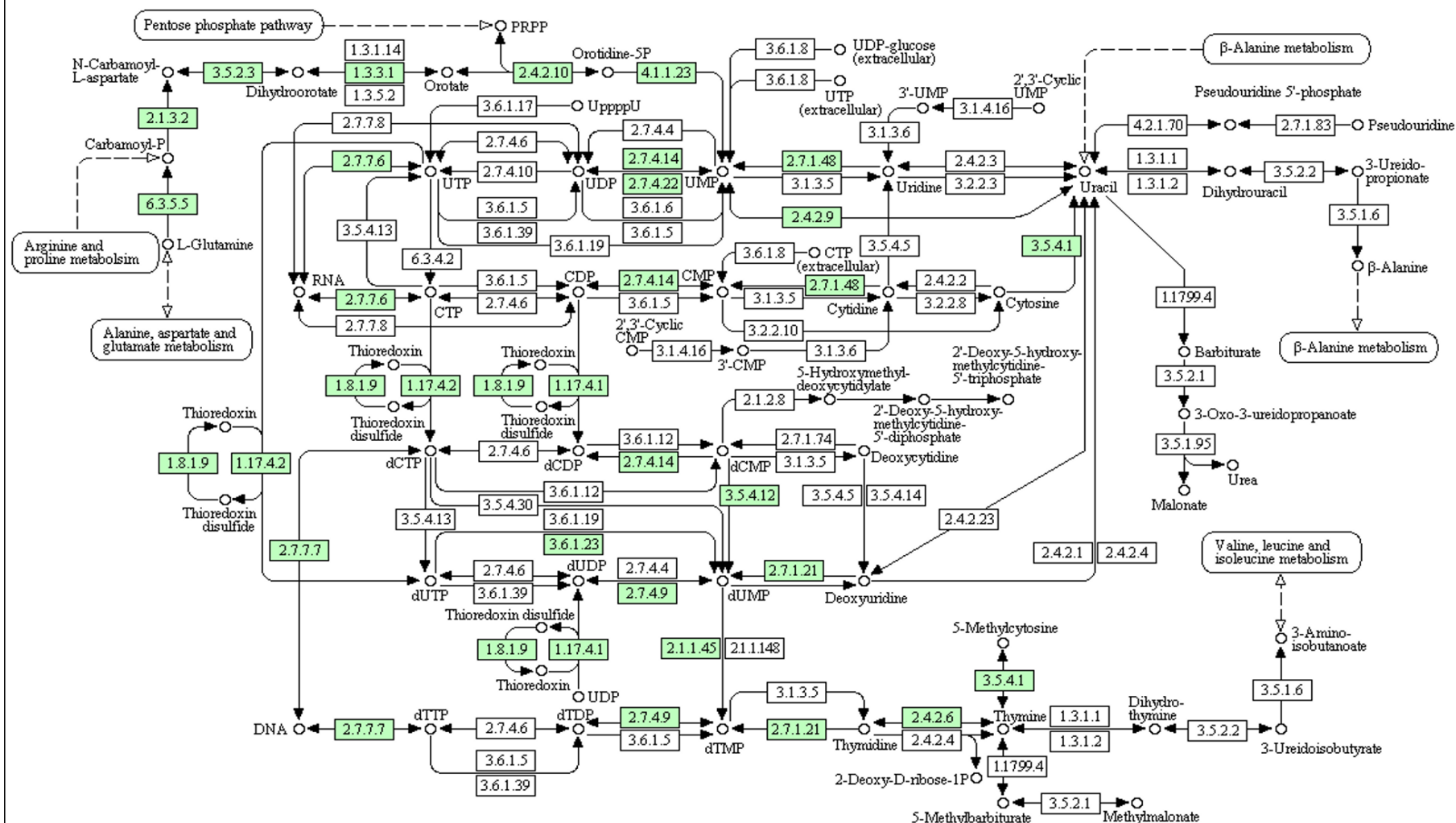

Supplement: Additional File 4 — Pyrimidine metabolism of L. ruminis ATCC 27782. Enzyme labels in green boxes represent those for which the corresponding gene was annotated in the genome. [file 1475-2859-10-S1-S13-S4.pdf]

# GLYCINE, SERINE AND THREONINE METABOLISM

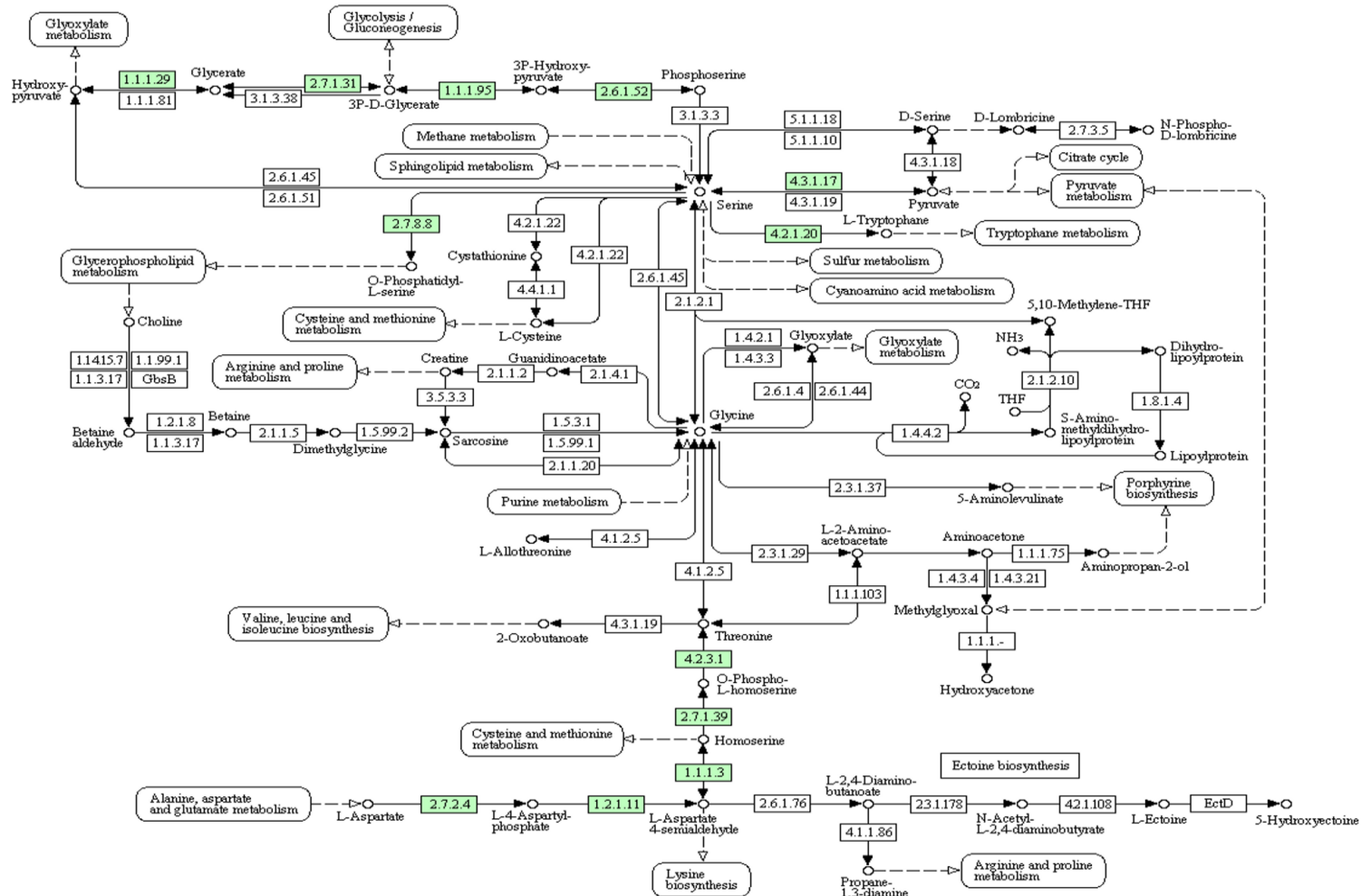

Supplement: Additional File 6 — Partial metabolic map of L. ruminis ATCC 27782, showing the predicted inter-conversions of pyruvate, serine, and tryptophan. Enzyme labels in green boxes represent those for which the corresponding gene was annotated in the genome. [file 1475-2859-10-S1-S13-S6.pdf]

# EPS Cluster

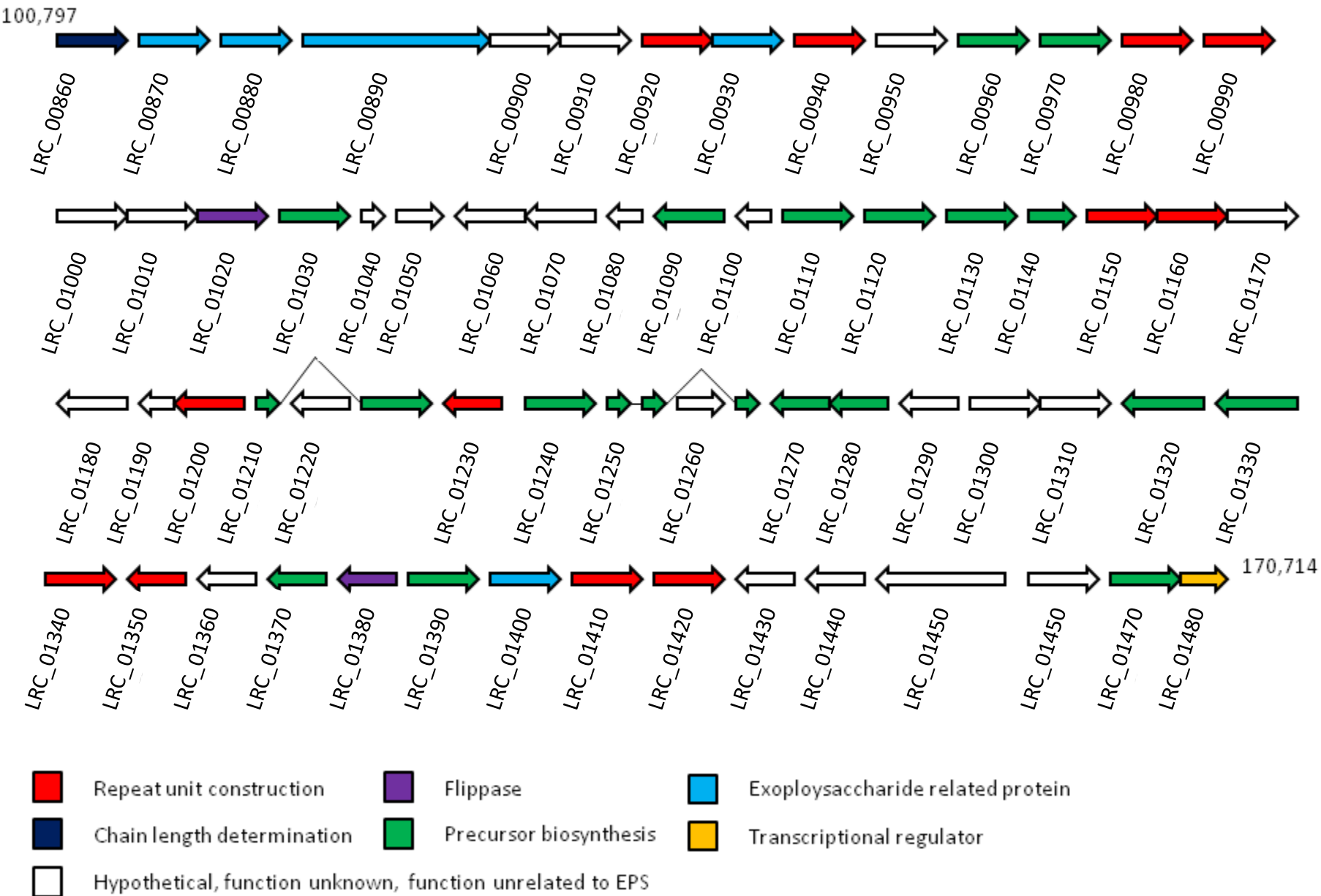

Supplement: Additional File 11 — Schematic diagram of a gene cluster predicted to encode EPS biosynthesis genes [file 1475-2859-10-S1-S13-S11.pdf]
