# Supplementary material for: Genome sequences and comparative genomics of two Lactobacillus ruminis strains from the bovine and human intestinal tracts
Source: Microb Cell Fact. 2011 Aug 30;10(Suppl 1):S13. doi: 10.1186/1475-2859-10-S1-S13 (PMC3231920; doi:10.1186/1475-2859-10-S1-S13)
Supplement: Additional File 7 — Schematic diagram of the locus encoding a putative Class IIa bacteriocin locus of L. ruminis ATCC 27782. Numbers above the diagram are nucleotide co-ordinates in the genome. Labels below the line are locus tags. [file 1475-2859-10-S1-S13-S7.pdf]

Bacteriocin locus

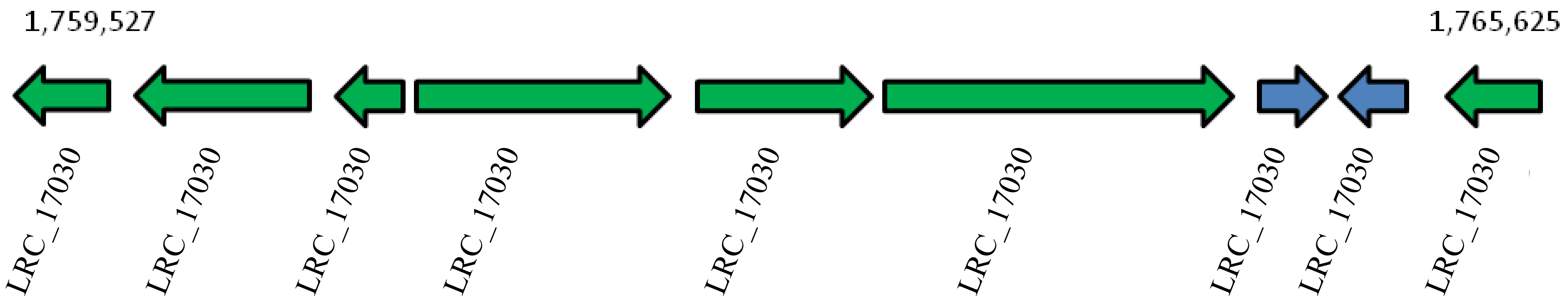

LRU_17030	Bacteriocin immunity protein
LRU_17040	Bacteriocin transport accessory protein
LRU_17050	Pediocin-like bacteriocin
LRU_17060	Sensor histidine protein kinase
LRU_17070	Response regulator
LRU_17080	Bacteriocin ABC transporter
LRU_17090	Hypothetical protein
LRU_17100	Hypothetical protein
LRU_17110	Bacteriocin immunity protein
