# Supplementary material for: Genome sequences and comparative genomics of two Lactobacillus ruminis strains from the bovine and human intestinal tracts
Source: Microb Cell Fact. 2011 Aug 30;10(Suppl 1):S13. doi: 10.1186/1475-2859-10-S1-S13 (PMC3231920; doi:10.1186/1475-2859-10-S1-S13)
Supplement: Additional File 8 — Multiple sequence alignment of the putative bacteriocin encoded by the LRC_17050 gene of L. ruminis ATCC 27782, and other Class II bacteriocin proteins, modified from Nissen-Meyer 2009, and Rea 2011 [46, 83]. Residues are numbered, by convention, with residue 1 being the first residue before the YGNG motif [46]. [file 1475-2859-10-S1-S13-S8.pdf]

Subgroup	Sequence
Subgroup 1	* (K position); ** (D position)
LRU_17050	KYGNGVYCGKHKCRVDWGQAWGCSVNRWGAAVGTGGKATIGHC
Coagulin	KYGNGVTCGKHSCSVDWGKATTCIINNGAMAWATGGHQGTHKC
Divergicin M35	KYGNGVYCNSKKOWVDWGTAQGCID--VVIGQLGGGIPGKGKC
Divergicin V41	KYGNGVYCNSKKOWVDWGQAHGCIGQTVVGGWLGGAIPG--KC
Enterocin A	TTHSGKYGNGVYCTKNKCTVDWAKATTCIAGMSIGGFLGGAIPG--KC
Leucocin C	KYGNGVHCTKKGCSVDWGYAWTNIANNSVMNGLTGGNAGWHN
Listeriocin 743 A	KSYGNGVHCNKKKCWVDWGSAISTIGNNSAANWATGGAAGWKS
Munticin	KYGNGVSCNKKGCSVDWGKAIGIIGNNSAANLATGGAAGWSK
Munticin KS	KYGNGVSCNKKGCSVDWGKAIGIIGNNSAANLATGGAAGWKS
Pediocin PA-1	KYGNGVTCGKHSCSVDWGKATTCIINNGAMAWATGGHQGNHKC
Pisciocin CS 526	KYGNGLSxNKKGxTVDWGTAIGIIGNNAAANxATGGAAGxNK
Piscicolin 126	KYGNGVSCNKNGCTVDWSKAIGIIGNNAAANLTTGGAAGWNKG
Sakacin P	KYGNGVHCGKHSCTVDWGTAIGNIGNNAAANWATGGNAGWNK
Sakacin 5X	KYGNGLSCNKSGCSVDWSKAISIIGNNAVANLTTGGAAGWKS
Subgroup II	**
Lactococcin MMFII	TSYGNGVHCNKSKOWIDVSELETYKAGTVSNPKDILW
Leucoccin A	YGNGVHCTKSGCSVNWGEAESAGVHRLANGGNGFW
Mesentericin Y105	YGNGVHCTKSGCSVNWGEAASAGIHRLANGGNGFW
Plantaricin C19	YGNGLSCSKKGCTVNWGQAFSCGVNRVATAGHGKx
Plantaricin 423	YGNGVTCGKHSCSVNWGQAFSCSVSHLANFGHGKC
Sakacin G	YGNGVSCNSHGCSVNWGQAWTCGVNHLANGGHGVC
Subgroup III	**
Curvicin A	RSYGNGVYCNNKKOWVNRGEATOSIIGGMISGWASGLAGM
Carnobacteriocin BM1	ISYGNGVYCNKEKOWVNKAENKQAITGIVIGGWASSLAQMGH
Enterocin P	ATRSYGNGVYCNNSKCWVNWGEAKENIAGIVISGWASGLAQMGH
Subgroup IV	**
Bacteriocin 31	TYGNGLYCNKOKOWVDWNKASREIGKIIVNGWVQHGPWAPR
Bacteriocin RC714	TYGNGLYCNKEKOWVDWNQAKGEIGKIIVNGWVNHGPWAP
Bacteriocin T8	TYGNGLYCNKEKOWVDWNQAKGEIGKIIVNGWVNHGPWAPRR
Penocin A	YGNGVHCGKKTOYVDWGQATASIGKIIVNGWTQHGPWAHR
Enterocin SE-K4	TYGNGVYCNKQKOWVDWSRARSEIIDRGVKAYVNGFTKVLG
Carnobacteriocin B2	VNYGNGVSCSKTKCSVNWGQAFQERYTAGINSFVSGVASGAGSIGRRP
