# Supplementary material for: Genome sequences and comparative genomics of two Lactobacillus ruminis strains from the bovine and human intestinal tracts
Source: Microb Cell Fact. 2011 Aug 30;10(Suppl 1):S13. doi: 10.1186/1475-2859-10-S1-S13 (PMC3231920; doi:10.1186/1475-2859-10-S1-S13)
Supplement: Additional file 9 — L. ruminis stress resistance proteins [file 1475-2859-10-S1-S13-S9.pdf]

**Locus tag**	**Start**	**Stop**	**Product**
LRC_02400	258534	258734	Cold shock protein
LRC_03340	356445	357338	Heat shock protein
LRC_03500	374410	374679	Heat shock protein
LRC_03590	382611	383498	Heat shock protein
LRC_06210	669979	670335	Phage shock protein C
LRC_08290	881675	882106	Alkaline shock protein
LRC_13160	1369008	1369217	Cold shock protein
LRC_19010	1970054	1970488	Heat shock protein
LRC_19270	2004650	2004907	Phage shock protein C
