# Supplementary material for: Genome sequences and comparative genomics of two Lactobacillus ruminis strains from the bovine and human intestinal tracts
Source: Microb Cell Fact. 2011 Aug 30;10(Suppl 1):S13. doi: 10.1186/1475-2859-10-S1-S13 (PMC3231920; doi:10.1186/1475-2859-10-S1-S13)
Supplement: Additional File 10 — L. ruminis sortase enzymes and sortase anchored proteins [file 1475-2859-10-S1-S13-S10.pdf]

Locus tag	Start	Stop	Product
LRC_00600	67416	70838	Sortase-anchored surface protein
LRC_00610	70969	71997	Sortase-anchored surface protein
LRC_00620	72000	73523	Sortase-anchored surface protein
LRC_00630	73657	74805	SrtC sortase
LRC_00700	81317	82801	Sortase-anchored surface protein
LRC_01690	189894	190127	Sortase-anchored surface protein
LRC_03170	334278	335657	Sortase-anchored surface protein
LRC_16530	1703432	1703566	Hypothetical protein
LRC_16570	1706763	1707416	SrtA sortase
LRC_16760	1735647	1736180	Sortase-anchored surface protein
LRC_16780	1736971	1737078	Hypothetical protein
LRC_16790	1737127	1737234	Hypothetical protein
