# Supplementary material for: Genome sequences and comparative genomics of two Lactobacillus ruminis strains from the bovine and human intestinal tracts
Source: Microb Cell Fact. 2011 Aug 30;10(Suppl 1):S13. doi: 10.1186/1475-2859-10-S1-S13 (PMC3231920; doi:10.1186/1475-2859-10-S1-S13)
Supplement: Additional File 12 — Annotation and phylogenetic relatedness of the EPS production locus of L. ruminis ATCC27782. [file 1475-2859-10-S1-S13-S12.pdf]

Locus tag	Start	Stop	Product	Species of BLAST top hit
LRC_00860	1	789	Exopolysaccharide chain length regulator	*Lactobacillus salivarius*
LRC_00870	807	1556	Tyrosine-protein kinase	*Lactobacillus salivarius*
LRC_00880	1556	2332	Phosphotyrosine-protein phosphatase	*Lactobacillus salivarius*
LRC_00890	2413	4422	Exopolysaccharide biosynthesis protein	*Coprococcus catus*
LRC_00900	4423	5262	HpcH/hpaI aldolase/citrate lyase family protein	*Eubacterium limosum*
LRC_00910	5275	6525	Diaminopimelate decarboxylase	*Bacteroides ovatus*
LRC_00920	6549	7241	Glycosyltransferase	*Butyrivibrio crossotus*
LRC_00930	7296	8225	Branched chain amino acid transaminase	*Bacteroides ovatus*
LRC_00940	8248	9054	Glycosyltransferase	*Butyrivibrio crossotus*
LRC_00950	9099	9929	Conserved hypothetical protein	*Ruminococcus sp. 18P13*
LRC_00960	9926	10753	NAD dependent epimerase	*Streptococcus iniae*
LRC_00970	10851	11432	Exoploysaccharide biosynthesis acetyltransferase	*bacterium Ellin514*
LRC_00980	11428	12339	Glycosyltransferase	*Paenibacillus polymyxa SC2*
LRC_00990	12374	13417	Glycosyltransferase	*Lactobacillus johnsonii*
LRC_01000	13444	14637	Hypothetical membrane protein	*Bacteroides sp. 3_1_19*
LRC_01010	14656	15627	Conserved hypothetical protein	*Clostridium acetobutylicum*
LRC_01020	15634	17115	Exoploysaccharide biosynthesis flippase enzyme	*Clostridium ramosum*
LRC_01030	17255	18205	Acetyltransferase	*Streptococcus anginosus*
LRC_01040	18342	18440	Hypothetical protein	*na*
LRC_01050	18627	18953	Transposase	*Lactobacillus crispatus*
LRC_01060	19048	19947	Conserved hypothetical protein	*Bacillus cereus*
LRC_01070	19954	21423	Conserved hypothetical protein	*Bacillus cereus*
LRC_01080	21477	21791	transposase	*Lactobacillus antri*
LRC_01090	22330	23811	Nucleotide sugar dehydrogenase	*Dorea longicatena*
LRC_01100	23924	24304	Hypothetical protien	*Mollicutes bacteriun*
LRC_01110	24615	25484	Glucose-1-phosphate Thymidylyltransferase	*Lactobacillus salivarius*
LRC_01120	25510	26529	dTDP-glucose 4,6-dehydratase	*Eubacterium ventriosum*
LRC_01130	26550	27464	dTDP-4-dehyrrorhamnose reductase	*Coprococcus eutactus*
LRC_01140	27479	28087	dTDP-4-dehydrorhamnose 3,5-epimerase	*Ruminococcus sp. 5_1_39B_FAA*
LRC_01150	29275	30306	Possible galactofuranosyltransferase	*Lactobacillus vaginalis*
LRC_01160	30307	31323	Possible galactofuranosyltransferase	*Lactobacillus fermentum*
LRC_01170	31348	32223	Conserved hypothetical protein	*Lactobacillus salivarius*
LRC_01180	32278	33144	DegV family protein	*Lactobacillus salivarius*
LRC_01190	33229	33642	GtrA containing transmembrane protein	*Ruminococcus lactaris*
LRC_01200	33655	34584	Glycosyltransferase	*Lactobacillus salivarius*
LRC_01210	34821	38869	Bacterial membrane protein	*Lactobacillus delbrueckii subsp bulgaricus*
LRC_01220	35449	36687	Transposase	*Collinsella stercoris*
LRC_01230	38853	39806	Glycosyltransferase	*Lactobacillus salivarius*
LRC_01240	40115	41572	Hypothetical membrane protein	*Lactobacillus reuteri*
LRC_01250	41989	43502	yfhO membrane protein	*Coprococcus sp. ART55/1*
LRC_01260	42551	42871	Transposon	*Atopobium vaginae*
LRC_01270	43578	44618	acyltransferase	*Atopobium vaginae*
LRC_01280	44611	46356	Hypothetical membrane protein	*Lactobacillus antri*
LRC_01290	46472	47959	Threonine synthase	*Lactobacillus salivarius*
LRC_01300	48149	49435	homoserine dehydrogenase	*Lactobacillus salivarius*
LRC_01310	49448	50320	homoserine kinase	*Lactobacillus salivarius*
LRC_01320	50355	51542	Hypothetical membrane protein	*Ruminococcus bromii*
LRC_01330	51656	53026	Cell wall binding repeat protein	*Lactobacillus salivarius*
LRC_01340	53293	54261	Glycosyltransferase	*Lactobacillus reuteri*
LRC_01350	54300	55220	Glycosyltransferase	*Lactobacillus oris*
LRC_01360	55222	56046	Hypothetical protein	*Lactobacillus vaginalis*
LRC_01370	56067	57269	Hypothetical membrane protein	*Lactobacillus salivarius*
LRC_01380	57262	58713	Oligosaccharide translocase	*Lactobacillus salivarius*
LRC_01390	58987	60102	UDP-galactopyranose mutase	*Lactobacillus salivarius*
LRC_01400	60129	60908	Possible capsular biosynthesis protein	*Pediococcus pentosaceus*
LRC_01410	60943	62394	Bacterial sugar transferase	*Ruminococcus torques*
LRC_01420	62454	63437	Glycosyltransferase	*Eubacterium rectale*
LRC_01430	63493	64095	Conserved hypothetical protein	*Lactobacillus brevis*
LRC_01440	64092	65303	Conserved hypothetical protein	*Lactobacillus brevis*
LRC_01450	65507	67378	Metal ion transport ATPase	*Lactobacillus salivarius*
LRC_01460	67668	68393	2-C-methyl-D-erythritol 4-phosphate cytidylyltransferase	*Clostridium sp. M62/1*
LRC_01470	68386	69447	NAD-dependent epimerase/dehydratase	*Coprococcus catus*

LRC_01480	69481	69918	Possible transcriptional regulator	*Oenococcus oeni*
