# Supplementary material for: Genome sequences and comparative genomics of two Lactobacillus ruminis strains from the bovine and human intestinal tracts
Source: Microb Cell Fact. 2011 Aug 30;10(Suppl 1):S13. doi: 10.1186/1475-2859-10-S1-S13 (PMC3231920; doi:10.1186/1475-2859-10-S1-S13)
Supplement: Additional File 13 — L. ruminis–specific proteins as determined by comparison with L. salivarius [file 1475-2859-10-S1-S13-S13.pdf]

Locus tag	Product	GC%
LRC_00180	MFS family major facilitator transporter	48.3
LRC_00220	Amidase	49.8
LRC_00290	Conserved hypothetical protein	41.9
LRC_00370	Hypothetical secreted protein	50.2
LRC_00470	Prolyl aminopeptidase	50.1
LRC_00480	Hypothetical membrane protein	46.8
LRC_00580	Hypothetical membrane protein	50.5
LRC_00600	Sortase-anchored surface protein	43.3
LRC_00610	Conserved Hypothetical sortase anchored surface eprotein	43.6
LRC_00630	Sortase	43.9
LRC_00760	AsnC family transcriptional regulator	48.6
LRC_00810	Toxin-antitoxin system, antitoxin component	39.6
LRC_00830	Conserved Hypothetical protein	32.2
LRC_00840	Conserved hypothetical protein	35.6
LRC_00910	Diaminopimelate decarboxylase	34.6
LRC_00930	Branched chain amino acid transaminase	35.7
LRC_00950	Conserved hypothetical protein	32.9
LRC_00960	NAD dependent epimerase	35.3
LRC_01000	Hypothetical membrane protein	28.4
LRC_01010	Conserved hypothetical protein	31.3
LRC_01030	Acetyltransferase	33.9
LRC_01060	Conserved hypothetical protein	31.9
LRC_01070	Conserved hypothetical protein	34.2
LRC_01190	GtrA containing transmembrane protein	41.1
LRC_01240	Hypothetical membrane protein	31.6
LRC_01270	acyltransferase	41.1
LRC_01350	Glycosyltransferase	34.6
LRC_01440	Conserved hypothetical protein	35.1
LRC_01460	2-C-methyl-D-erythritol 4-phosphate cytidylyltransferase	44.5
LRC_01470	NAD-dependent epimerase/dehydratase	42.6
LRC_01510	Conserved hypothetical protein	42.7
LRC_01580	Signal transduction histidine kinase	46.4
LRC_01670	conserved hypothetical protein	45.3
LRC_01780	oligopeptide ABC superfamily ATP binding cassette transporter,binding protein	43.6
LRC_01820	Guanylate kinase	47
LRC_02040	Exopolysaccharide glycosyltransferase	43.3
LRC_02140	Cation transporter	46.8
LRC_02200	transcriptional regulator	44
LRC_02240	Cellobiose PTS, EIIC	44.4
LRC_02250	cellobiose PTS, EIIB	41.5
LRC_02260	PTS family lactose/cellobiose porter component IIA	41.8
LRC_02280	ROK family sugar kinases	44.6
LRC_02370	Hypothetical membrane protein	41.2
LRC_02510	Hypothetical membrrane protein	43.1
LRC_02520	Rrf2 family transcriptional regulator	50.5
LRC_02700	ATP-grasp domain protein	40.6
LRC_02720	Hypothetical protein	37.8
LRC_03100	Possible Lysophospholipase	46.9
LRC_03150	Hypothetical deoxyribonuclease	45.3
LRC_03420	Hypothetical protein	41.8
LRC_03450	IstB ATP binding domain-containing protein	41.6
LRC_03650	Hypothetical protein	43.2

LRC_03670	Hypothetical protein	40.1
LRC_03680	Conserved hypothetical protein	32.6
LRC_03900	FIST domain containing protein	43.6
LRC_04200	Glutamate synthase large subunit	46
LRC_04280	Hypothetical protein	42.6
LRC_04340	Hypothetical membrane protein	38.9
LRC_04360	Integrase catalytic region	47.4
LRC_04490	TetR transcriptional regulator	38.3
LRC_04620	Hypothetical protein	36.7
LRC_04910	3-deoxy-7-phosphoheptulonate synthase	49.9
LRC_05120	Conserved hyothetical protein	46.3
LRC_05160	Integrase	45.2
LRC_05180	Conserved hypothetical protein	35.8
LRC_05240	IstB ATP binding domain-containing protein	41.9
LRC_05250	integrase	38.6
LRC_05280	Hypothetical protein	43
LRC_05290	Hypothetical protein	40.2
LRC_05310	SNF2 family DNA/RNA helicases	54.3
LRC_05320	Quinolinate synthetase A	39.4
LRC_05330	L-aspartate oxidase	42.1
LRC_05340	Nicotinate-nucleotide diphosphorylase	42.3
LRC_05370	Putative methylase	42.5
LRC_05400	Hypothetical protein	37.8
LRC_05410	Toxin/antitoxin system, Toxin component	36
LRC_05420	Toxin/antitoxin system, Antitoxin component	40.6
LRC_05510	MFS transporter	47.4
LRC_05530	Acetyltransferase	47.7
LRC_05580	Hypothetical membrane protein	49
LRC_05610	Alcohol dehydrogenase	57.3
LRC_05680	Permease of the major facilitator superfamily	51.7
LRC_05690	ABC transporter ATP-binding protein	51.5
LRC_05890	Conserved hypothetical protein	45.8
LRC_05940	Aconitate hydratase	47.8
LRC_05960	Isocitrate dehydrogenase	46.9
LRC_06150	Cell-division associated ABC transporter, membrane FtsX subunit	44
LRC_06170	Flagellin	44.9
LRC_06350	Hypothetical membrane protein	36.8
LRC_06510	GNAT family N-acetyltransferase	45.6
LRC_06600	Histidine-tRNA ligase	43.5
LRC_06610	ATP phosphoribosyltransferase	46.9
LRC_06630	Imidazoleglycerol-phosphate dehydratase	45
LRC_06640	Imidazole glycerol phosphate synthase, glutamine amidotransferase subunit	43.1
LRC_06650	1-(5-phosphoribosyl)-5-[(5-phosphoribosylamino)methylideneamino]imidazole-4-carboxamide isomerase	46
LRC_06660	Imidazoleglycerol phosphate synthase, cyclase subunit	45.5
LRC_06690	Histidinol-phosphate aminotransferase	41.8
LRC_06700	GNAT family acetyltransferase	43.4
LRC_06800	LysM-domain protein/autolysin	39.7
LRC_06910	putative ion Mg(2+)/C(o2+) transport protein	43.5
LRC_07190	Possible dnaD domain protein	46.3
LRC_07210	Possible trasposase	46.8
LRC_07250	Conserved hypothetical protein/possible phage	37.5
LRC_07260	Phage terminase	46.4

LRC_07270	Phage protein	45.7
LRC_07280	Phage structural protein	48.6
LRC_07320	Phage protein	39.4
LRC_07340	Phage protein	38.4
LRC_07370	Phage tail protein	44
LRC_07430	Lysin	44.4
LRC_07440	Conserved hypothetical phage protein	32.9
LRC_07540	Fe2+ transport system protein A	48.5
LRC_07550	Fe2+ transport system protein B	46.3
LRC_07560	Hypothetical protein	35.2
LRC_07810	Peptidase	39.6
LRC_07910	Anthranilate synthase component II	41.4
LRC_07960	tryptophan synthase, alpha subunit	42.4
LRC_07970	Hypothetical PAP2 superfamily protein	40.9
LRC_07990	phosphatidylserine decarboxylase fragment	38.7
LRC_08010	Conserved hypothetical protein	39.5
LRC_08040	hypothetical protein	38.8
LRC_08050	integrase catalytic subunit	47.5
LRC_08110	Transcriptional regulator, GntR family	41.7
LRC_08180	conserved hypothetical protein	40.7
LRC_08240	ABC superfamily ATP binding cassette transporter, substrate binding protein	45.4
LRC_08410	multidrug ABC transporter ATP-binding and permease component	43.4
LRC_08550	Hypothetical protein	41.5
LRC_08670	transcriptional regulator, GntR family with aminotransferase domain	46.9
LRC_08810	type III restriction-modification system	35.2
LRC_08830	Conserved hypothetical protein	36.9
LRC_08840	SNF2 family helicase	38.2
LRC_08850	conserved hypothetical protein	43.8
LRC_08900	putative HNH endonuclease, phage-related	42.1
LRC_08930	conserved hypothetical protein	32.8
LRC_08940	Putative phage-associated protein	37.6
LRC_08950	conserved hypothetical protein	40.8
LRC_08960	conserved hypothetical protein	44.9
LRC_09190	Hypothetical protein	35.6
LRC_09380	putative ATPase (PilT family)	42.8
LRC_09470	pyrophosphohydrolase, NUDIX family	39.6
LRC_09620	major facilitator superfamily permease	48.6
LRC_09740	hypothetical membrane protein	43.2
LRC_09810	MATE efflux family protein	46.5
LRC_09830	methyl-accepting chemotaxis sensory transducer	44.4
LRC_09850	putative transcriptional regulator	40.7
LRC_09860	CRP/FNR family transcriptional regulator	40.9
LRC_09870	conserved hypothetical protein	44.9
LRC_09940	CRISPR-associated Csm5 family protein	38.1
LRC_09960	CRISPR-associated RAMP protein, csm3 family	43.6
LRC_09970	CRISPR-associated protein, Csm2 family	42
LRC_09980	CRISPR-associated Csm1 family protein	47.6
LRC_09990	CRISPR-associated RAMP superfamily protein	45.2
LRC_10010	hypothetical protein	28.6
LRC_10200	CRISPR-associated protein Cas4	26.2
LRC_10220	CRISPR-associated protein Cas5	32.7
LRC_10230	crispr-associated regulatory protein, devr family	33.4
LRC_10240	CRISPR-associated CXXC_CXXC protein Cst1	28.2

LRC_10250	putative crispr-associated protein cas6	31.7
LRC_10270	putative DNA endonuclease	45.3
LRC_10420	SNF2 family helicase	42.1
LRC_10630	Hypothetical protein	34.3
LRC_10700	Hypothetical protein	41.1
LRC_10730	Methyltyransferase	42.2
LRC_10930	Conserved hypothetical protein, AraC domain	41.4
LRC_11230	MATE efflux family protein	43.3
LRC_11360	Transcriptional regulator	42
LRC_11450	Integrase, catalytic region	47.1
LRC_11570	AP endonuclease	45.5
LRC_11580	conserved secreated protein	49.8
LRC_11610	Core-2/I-Branching enzyme	43.1
LRC_11890	Conserved hypothetical protein	42.2
LRC_11970	gedit	41.3
LRC_12180	serine/threonine protein kinase	43.8
LRC_12540	hypothetical protein	34.3
LRC_12660	conserved hypothetical protein	40.2
LRC_12970	UDP-N-acetylmuramoylalanyl-D-glutamate--2,6-diaminopimelate ligase	42.8
LRC_13020	conserved hypothetical protein	33.3
LRC_13280	Conserved hypothetical protein	42.5
LRC_13870	Hypothetical protein	48.1
LRC_14260	metallophosphoesterase	43.3
LRC_14280	Transcriptional regulator	41.7
LRC_14350	Conserved hypothetical protein	38.6
LRC_14380	Hypothetical protein, possible membrane protein	38.5
LRC_14520	major facilitator superfamily MFS_1	43
LRC_14570	Asparagine synthase	43.7
LRC_14620	Hypothetical protein	40.3
LRC_14650	Conserved hypothetical protein	41.4
LRC_14750	Hypothetical protein	42.5
LRC_14800	Hypothetical protein	43.8
LRC_14850	Oxidoreductase family, NAD-binding Rossmann fold protein	46.1
LRC_14990	Integrase catalytic region	48.1
LRC_15090	Ferritin, Dps family protein	42.1
LRC_15100	Hypothetical protein	35.8
LRC_15120	Hypothetical protein	34.7
LRC_15130	Hypothetical protein	35.5
LRC_15150	Conserved hypothetical protein	37.7
LRC_15410	Nitroreductase	45.8
LRC_15460	Methyl accepting chemotaxis protein	46.2
LRC_15470	Conserved hypothetical protein	31
LRC_15510	ABC transporter permease	37.5
LRC_15560	Glycerol-3-phosphate cytidylyltransferase	43.5
LRC_15580	Aminotransferase class V	44.7
LRC_15610	NAD/NADP octopine/nopaline dehydrogenase	36.9
LRC_15620	Glycosyltransferase family 2	39.8
LRC_15670	Glycosyl transferase family 2	37
LRC_15720	Conserved hypothetical protein	40.2
LRC_15740	Flagellar hook associated protein	46.1
LRC_15750	Flagellar hook associated protein	47.8
LRC_15770	Negative regulator of flagellin synthesis	42.7
LRC_15780	Flagellar motor switch protein	50.5
LRC_15830	Chemotaxis protein, cheA	46.2

LRC_15860	Chemotaxis protein, CheD	48.9
LRC_15870	Chemotaxis protein, cheW	39.9
LRC_15880	Methyl-accepting chemotaxis sensory transducer	46.9
LRC_15900	Flagellar basal-body rod protein	49.2
LRC_15920	RNA polymerase, sigma-factor 28	43
LRC_15930	flagellar biosynthesis protein FlhA	46.7
LRC_15940	flagellar biosynthetic protein flhB	46.8
LRC_15960	Flagellar biosynthesis protein, FliQ	41.7
LRC_15990	flagellar biosynthesis protein, FliL	46.8
LRC_16020	Flagellar operon protein	39.9
LRC_16030	Flagellar basal body rod modification protein	45.6
LRC_16040	Flagellar hook-length control protein, FliK	48.2
LRC_16110	Flagellar basal-body rod protein, FlgC	42.4
LRC_16140	Flagellar motor protein, MotB	48.8
LRC_16170	Methyl accepting chemotaxis protein	46.6
LRC_16190	Conserved hypothetical protein	34.7
LRC_16230	Putative membrane-associated hydrolase	43.2
LRC_16240	Hypothetical protein	43
LRC_16280	Conserved hypothetical secreted protein	41.5
LRC_16290	Hypothetical protein	43.1
LRC_16460	Hypothetical membrane protein	44.7
LRC_16490	Putative permease	45.7
LRC_16510	Hypothetical protein	30.6
LRC_16530	hypothetical protein	41.5
LRC_16620	HTH containing DNA-binding domain and MocR-like aminotransferase	46.6
LRC_16700	hydrolase	47
LRC_16730	Hypothetical secreted protein	39.4
LRC_16740	two-component sensor histidine kinase	38.9
LRC_16810	ABC transporter protein	45.7
LRC_16830	ABC transporter permease	47.5
LRC_16840	ABC transporter, permease protein	46.9
LRC_16920	glycerophosphodiester phosphodiesterase	44.2
LRC_16950	ABC transporter, permease protein	45.4
LRC_17030	potential bacteriocin immunity protein	47.2
LRC_17040	bacterocin transport accessory protein	48.1
LRC_17050	putative bacteriocin	54.4
LRC_17230	amidinotransferase family protein	47.8
LRC_17250	putative dehydrogenase	45.4
LRC_17320	ABC transporter, permease	47.2
LRC_17360	TetR family transcriptional regulator	38.6
LRC_17400	ABC transporter, permease	45
LRC_17410	Major facilitator family transporter	31.1
LRC_17540	ABC transporter substrate-binding component	46
LRC_17590	TetR family transcriptional regulator	38.7
LRC_17610	Hypothetical protein	41.3
LRC_17650	pseudogene - efflux protein	50.3
LRC_17670	pseudogene - efflux protein	46.2
LRC_17820	Conserved hypothetical membrane protein - cellobiose PTS	42.9
LRC_17830	Cellobiose PTS IIc component	48.3
LRC_17840	Conserved hypothetical flavodoxin-fold protein	42.6
LRC_17890	acetylglutamate kinase	47.7
LRC_17910	N-acetyl-gamma-glutamyl-phosphate reductase	44.6
LRC_17940	Hypothetical protein	47.1
LRC_17950	Hypothetical efflux protein	47

LRC_17960	Hypothetical hydrolase	45.2
LRC_18020	conserved hypothetical protein	42.4
LRC_18060	Hydrolase	44
LRC_18090	conserved hypothetical protein	41.7
LRC_18110	conserved hypothetical protein	45.7
LRC_18150	Hypothetical protein	38.2
LRC_18180	Hypothetical protein	48.2
LRC_18210	Conserved hypothetical protein	38.4
LRC_18260	AraC family transcriptional regulator	47.6
LRC_18340	ABC superfamily ATP binding cassette transporter permease protein	47.1
LRC_18380	Conserved hypothetical protein	44.4
LRC_18390	Response regulator of the LytR/AlgR family protein	40.6
LRC_18410	Helix-turn-helix, Fis family protein	42.9
LRC_18430	Aspartate kinase	46.3
LRC_18510	Lipase/esterase	47.5
LRC_18550	Hypothetical protein	42.3
LRC_18720	Maltose/maltodextrin ABC superfamily ATP binding cassette transporter, permease protein	45.5
LRC_18730	Maltose/maltodextrin ABC superfamily ATP binding cassette transporter, permease protein	42.4
LRC_18740	Maltose/maltodextrin ABC superfamily ATP binding cassette transporter, binding protein	44.3
LRC_18770	LacI family transcriptional regulator	44.4
LRC_18810	Endonuclease/exonuclease/phosphatase family protein	44.2
LRC_18900	Transcriptional regulator	44.7
LRC_18920	Ubiquinone biosynthesis protein	45
LRC_18950	Prephenate dehydrogenase	48.2
LRC_18960	3-phosphoshikimate 1-carboxyvinyltransferase	51.4
LRC_18980	Chorismate synthase	53.5
LRC_19000	Multidrug ABC superfamily ATP binding cassette transporter, ATPase and permease protein	45.1
LRC_19010	Heat shock protein Hsp20	43.9
LRC_19080	ABC-type sugar transport system, periplasmic component	44.5
LRC_19090	Methyl-accepting chemotaxis protein, contain HAMP domain	44.4
LRC_19120	Endonuclease/exonuclease/phosphatase	43.5
LRC_19170	ABC superfamily, ATP binding cassette transporter, membrane protein	48.4
LRC_19200	ABC superfamily ATP binding cassette transporter, binding protein	48.9
LRC_19280	Conserved hypothetical protein	45.9
LRC_19320	Haloacid dehalogenase superfamily hydrolase	44.5
LRC_19390	Integrase catalytic region	46.7
LRC_19400	Major facilitator transporter	51.1
LRC_19410	Major facilitator family transporter	31.1
LRC_19450	Conserved hypothetical membrane protein	45.4
LRC_19590	6-phospho-beta-glucosidase	48.5
LRC_19610	PTS system, lactose/cellobiose-specific IIA subunit	45.2
LRC_19640	6-phospho-beta-glucosidase	48
LRC_19750	Cobalt ABC superfamily ATP binding cassette transporter permease	42.9
LRC_19760	Conserved hypothetical membrane protein	48.7
