# Supplementary material for: Genome sequences and comparative genomics of two Lactobacillus ruminis strains from the bovine and human intestinal tracts
Source: Microb Cell Fact. 2011 Aug 30;10(Suppl 1):S13. doi: 10.1186/1475-2859-10-S1-S13 (PMC3231920; doi:10.1186/1475-2859-10-S1-S13)
Supplement: Additional File 14 — L. salivarius-specific proteins as determined by comparison with L. ruminis [file 1475-2859-10-S1-S13-S14.pdf]

| Locus tag | Products |
|---|---|
| LSL_0010 | 3-hydroxyisobutyrate dehydrogenase |
| LSL_0013 | Large-conductance mechanosensitive channel |
| LSL_0014 | Pyruvate oxidase |
| LSL_0016 | UDP-N-acetylmuramoyl-L-alanyl-D-glutamate-- lysine ligase |
| LSL_0017 | Conserved hypothetical protein |
| LSL_0018 | Aspartate racemase |
| LSL_0020 | Transcriptional regulator, TetR family |
| LSL_0029 | Peptide methionine sulfoxide reductase |
| LSL_0030 | DNA-damage-inducible protein J |
| LSL_0044 | Oxidoreductase |
| LSL_0047 | Conserved hypothetical protein |
| LSL_0048 | Glycerol-3-phosphate transporter |
| LSL_0052 | Na+ driven multidrug efflux pump |
| LSL_0053 | ABC transporter, ATP-binding protein |
| LSL_0056 | Glutamine amidotransferase, class I |
| LSL_0057 | Glutamate--cysteine ligase |
| LSL_0061 | Hypothetical protein |
| LSL_0070 | Putative Zn-dependent protease |
| LSL_0071 | N-acetylglucosaminyltransferase |
| LSL_0077 | Conserved hypothetical protein |
| LSL_0080 | Glyoxalase family protein |
| LSL_0083 | Two-component response regulator |
| LSL_0085 | D-ribose-binding protein |
| LSL_0097 | Hypothetical protein |
| LSL_0107 | Hypothetical protein |
| LSL_0110 | Glycerol kinase |
| LSL_0126 | CAAX amino terminal protease family |
| LSL_0127 | Flavodoxin |
| LSL_0133 | Succinyl-diaminopimelate desuccinylase |
| LSL_0134 | NAD(P)H-dependent quinone reductase |
| LSL_0136 | 3-hydroxybutyryl-CoA dehydrogenase |
| LSL_0137 | NADH peroxidase |
| LSL_0138 | Ornithine decarboxylase |
| LSL_0139 | Hypothetical protein |
| LSL_0142 | Conserved hypothetical protein |
| LSL_0147 | 2,5-diketo-D-gluconic acid reductase |
| LSL_0148 | Short chain dehydrogenase |
| LSL_0149 | Hypothetical protein |
| LSL_0154 | Pyruvate dehydrogenase E1 component beta subunit |
| LSL_0156 | Dihydrolipoamide dehydrogenase |
| LSL_0157 | Lipoate-protein ligase A |
| LSL_0159 | Hypothetical protein |
| LSL_0174 | Naphthoate synthase |
| LSL_0175 | O-succinylbenzoic acid--CoA ligase |
| LSL_0183 | Transcriptional regulators, LysR family |

| | |
|---|---|
| LSL_0184 | Phosphatase |
| LSL_0185 | Acetyltransferase, GNAT family |
| LSL_0186 | Acetolactate synthase |
| LSL_0204 | Aminotransferase class I and II |
| LSL_0208 | Hypothetical membrane spanning protein |
| LSL_0209 | 1-deoxy-D-xylulose 5-phosphate synthase |
| LSL_0221 | ABC transporter permease protein |
| LSL_0222 | Transcriptional regulator, TetR family |
| LSL_0225 | IpaB/EvcA family protein |
| LSL_0231 | Conserved hypothetical protein |
| LSL_0232 | aminotransferase |
| LSL_0236 | Phage integrase |
| LSL_0238 | Hypothetical protein, phage associated |
| LSL_0240 | cII-like protein, phage associated |
| LSL_0241 | Hypothetical protein, phage associated |
| LSL_0250 | Cro-like protein, phage associated |
| LSL_0251 | Phage antirepressor protein |
| LSL_0252 | Hypothetical protein, phage associated |
| LSL_0256 | Hypothetical protein, phage associated |
| LSL_0257 | Replisome organizer, phage associated |
| LSL_0259 | Hypothetical protein, phage associated |
| LSL_0266 | Hypothetical protein, phage associated |
| LSL_0276 | Hypothetical protein, phage associated |
| LSL_0277 | DNA packaging, phage associated |
| LSL_0280 | Phage Terminase Small Subunit |
| LSL_0281 | Terminase large subunit |
| LSL_0283 | Portal protein |
| LSL_0288 | Phage head-tail joining protein |
| LSL_0289 | Phage tail protein |
| LSL_0290 | Phage major tail protein |
| LSL_0291 | Hypothetical protein, phage associated |
| LSL_0294 | phage tail tape mesure |
| LSL_0296 | Phage lysin |
| LSL_0299 | Hypothetical protein, phage associated |
| LSL_0302 | Hypothetical protein, phage associated |
| LSL_0304 | Phage lysin |
| LSL_0309 | Hypothetical membrane spanning protein |
| LSL_0316 | Macrolide-efflux protein |
| LSL_0319 | Ribosomal-protein-serine acetyltransferase |
| LSL_0320 | 2,5-diketo-D-gluconic acid reductase |
| LSL_0330 | Hypothetical protein |
| LSL_0334 | Hypothetical secreted protein |
| LSL_0336 | Hypothetical membrane spanning protein |
| LSL_0351 | Hypothetical secreted protein |
| LSL_0365 | Conserved hypothetical protein |
| LSL_0410 | Hypothetical membrane spanning protein |

| | |
|---|---|
| LSL_0427 | Hypothetical protein |
| LSL_0431 | Hypothetical protein |
| LSL_0476 | Hypothetical protein |
| LSL_0482 | Conserved hypothetical protein |
| LSL_0518 | Choloylglycine hydrolase |
| LSL_0519 | Hypothetical protein |
| LSL_0545 | Hypothetical membrane spanning protein |
| LSL_0547 | Hypothetical protein |
| LSL_0608 | Arsenate reductase |
| LSL_0650 | Lactate/malate dehydrogenase |
| LSL_0695 | Glycosyltransferase |
| LSL_0698 | Hypothetical membrane spanning protein |
| LSL_0713 | Hypothetical protein |
| LSL_0723 | Transcriptional regulator, TetR family |
| LSL_0725 | Hypothetical ABC transporter permease protein |
| LSL_0740 | Phage super infection exclusion |
| LSL_0746 | Phage antirepressor |
| LSL_0751 | Hypothetical protein, phage associated |
| LSL_0752 | Phage helicase |
| LSL_0756 | Phage NTP-binding protein |
| LSL_0758 | Phage DNA polymerase |
| LSL_0764 | Hypothetical protein, phage associated |
| LSL_0765 | Phage integrase |
| LSL_0768 | Hypothetical protein, phage associated |
| LSL_0770 | Hypothetical protein, phage associated |
| LSL_0773 | Hypothetical protein, phage associated |
| LSL_0775 | Hypothetical protein, phage associated |
| LSL_0778 | Hypothetical protein, phage associated |
| LSL_0783 | Terminase large subunit |
| LSL_0784 | Portal protein |
| LSL_0788 | Phage head-tail joining protein |
| LSL_0790 | Phage head-tail joining protein |
| LSL_0797 | Phage lysin |
| LSL_0798 | Hypothetical protein, phage associated |
| LSL_0799 | Phage minor head protein |
| LSL_0803 | Hypothetical protein, phage associated |
| LSL_0804 | Hypothetical protein, phage associated |
| LSL_0805 | Phage lysin |
| LSL_0807 | Hypothetical protein |
| LSL_0808 | Hypothetical protein |
| LSL_0828 | Uracil permease |
| LSL_0850 | Transporter, drug/metabolite exporter family |
| LSL_0854 | Magnesium and cobalt transport protein corA |
| LSL_0855 | Na(+)/H(+) antiporter |
| LSL_0869 | Hypothetical membrane spanning protein |
| LSL_0874 | Transcriptional regulator, MarR family |

| | |
|---|---|
| LSL_0879 | Phosphoesterase |
| LSL_0880 | Transporter, drug/metabolite exporter family |
| LSL_0883 | Conserved hypothetical protein |
| LSL_0903 | Conserved hypothetical protein |
| LSL_0906 | Hypothetical membrane spanning protein |
| LSL_0917 | DNA integration/recombination/inversion protein |
| LSL_0918 | Type I restriction-modification system specificity subunit |
| LSL_0919 | Type I restriction-modification system methylation subunit |
| LSL_0921 | Hypothetical protein |
| LSL_0945 | Hypothetical protein |
| LSL_0953 | Hypothetical protein |
| LSL_0958 | Hypothetical protein |
| LSL_0961 | Hypothetical protein |
| LSL_0962 | Hypothetical protein |
| LSL_0963 | Hypothetical protein |
| LSL_0965 | UDP-glucose 4-epimerase |
| LSL_0966 | Acetyltransferase |
| LSL_0967 | Hypothetical protein |
| LSL_0968 | Hypothetical membrane spanning protein |
| LSL_0969 | Hypothetical protein |
| LSL_0973 | Conserved hypothetical protein |
| LSL_0975 | Hypothetical membrane spanning protein |
| LSL_0977 | Glycosyltransferase |
| LSL_0978 | Glycosyltransferase |
| LSL_0980 | UDP-glucuronate 4-epimerase |
| LSL_0981 | Oligosaccharide translocase |
| LSL_0982 | Glycosyltransferase |
| LSL_0983 | Glycosyltransferase |
| LSL_0986 | Glycosyltransferase |
| LSL_0987 | Glycosyltransferase |
| LSL_0988 | Glycosyltransferase |
| LSL_0989 | Glycosyltransferase |
| LSL_0990 | Glycosyltransferase |
| LSL_0991 | Glycosyltransferase |
| LSL_0992 | Acetyltransferase |
| LSL_0997 | Chain length regulator |
| LSL_1003 | Acetyltransferase |
| LSL_1008 | Transcriptional regulator, AraC family |
| LSL_1016 | Conserved hypothetical protein |
| LSL_1018 | Cellulose synthase catalytic subunit |
| LSL_1020 | Conserved hypothetical protein |
| LSL_1023 | Conserved hypothetical protein |
| LSL_1024 | Response regulator |
| LSL_1026 | NADH dehydrogenase |
| LSL_1028 | Farnesyl pyrophosphate synthetase |
| LSL_1029 | Transport ATP-binding protein |

| | |
|---|---|
| LSL_1030 | Transport ATP-binding protein |
| LSL_1033 | Aspartate--ammonia ligase |
| LSL_1035 | Rhodanese-related sulfurtransferases |
| LSL_1039 | Hypothetical protein |
| LSL_1040 | ADP-ribosylglycohydrolase |
| LSL_1057 | Hypothetical membrane spanning protein |
| LSL_1085 | Hypothetical surface protein |
| LSL_1086 | Hypothetical membrane spanning protein |
| LSL_1087 | Conserved hypothetical protein |
| LSL_1091 | Single-stranded-DNA-specific exonuclease |
| LSL_1099 | Glycerate dehydrogenase |
| LSL_1115 | Conserved hypothetical protein |
| LSL_1116 | iron-sulfur cluster-binding protein |
| LSL_1117 | Cysteine-rich domain of 2-hydroxy-acid oxidase GlcF |
| LSL_1119 | Hypothetical protein |
| LSL_1137 | 23S rRNA m(1)G 745 methyltransferase |
| LSL_1140 | Proton/sodium-glutamate symport protein |
| LSL_1172 | Oxidoreductase |
| LSL_1187 | Transporter, MFS superfamily |
| LSL_1189 | Phage integrase |
| LSL_1190 | Hypothetical protein, phage associated |
| LSL_1195 | Hypothetical protein, phage associated |
| LSL_1196 | Hypothetical protein, phage associated |
| LSL_1197 | Hypothetical protein, phage associated |
| LSL_1202 | DNA primase |
| LSL_1203 | Hypothetical protein, phage associated |
| LSL_1217 | Ribosomal-protein-S18-alanine acetyltransferase |
| LSL_1236 | Hypothetical protein |
| LSL_1267 | Hypothetical membrane spanning protein |
| LSL_1275 | Transcriptional regulator |
| LSL_1290 | Glycogen phosphorylase |
| LSL_1293 | Glucose-1-phosphate adenylyltransferase catalytic subunit |
| LSL_1296 | Conserved hypothetical protein |
| LSL_1315 | Transcriptional regulator, MarR family |
| LSL_1322 | Alkaline phosphatase |
| LSL_1325 | 2-oxoglutarate/malate translocator |
| LSL_1329 | Pyridine nucleotide-disulphide oxidoreductase family protein |
| LSL_1334 | Acetyltransferase |
| LSL_1335 | Mucus binding protein |
| LSL_1336 | Acetyltransferase |
| LSL_1372 | Pyruvate oxidase |
| LSL_1389 | Hypothetical protein |
| LSL_1390 | Hypothetical protein |
| LSL_1391 | Hypothetical protein |
| LSL_1394 | Hypothetical protein |
| LSL_1396 | Hypothetical protein |

| | |
|---|---|
| LSL_1397 | Dehydrogenase |
| LSL_1401 | Conserved hypothetical protein |
| LSL_1442 | Amino acid permease |
| LSL_1445 | Glutamine-binding protein |
| LSL_1447 | Conserved hypothetical protein |
| LSL_1462 | Na+ driven multidrug efflux pump |
| LSL_1467 | Succinyl-diaminopimelate desuccinylase |
| LSL_1483 | Hypothetical membrane spanning protein |
| LSL_1493 | Hypothetical membrane spanning protein |
| LSL_1502 | Putative phosphatase |
| LSL_1504 | FMN reductase |
| LSL_1513 | Trehalose operon transcriptional repressor |
| LSL_1523 | Hypothetical membrane spanning protein |
| LSL_1524 | Glycosyltransferase |
| LSL_1527 | O-acetyl transferase |
| LSL_1529 | Hypothetical membrane spanning protein |
| LSL_1536 | Hypothetical membrane spanning protein |
| LSL_1542 | Amino acid permease |
| LSL_1543 | Conserved hypothetical protein |
| LSL_1544 | Multidrug resistance efflux pump |
| LSL_1555 | Polysacharide biosyntheis protein |
| LSL_1558 | Capsular polysaccharide synthesis protein |
| LSL_1559 | Glycosyltransferase |
| LSL_1561 | Glycosyltransferase |
| LSL_1564 | DNA helicase |
| LSL_1573 | Glycosyltransferase |
| LSL_1578 | Multidrug resistance protein B |
| LSL_1581 | Transcriptional regulator, MerR family |
| LSL_1615 | Hypothetical protein |
| LSL_1616 | ABC transporter, ATP-binding protein |
| LSL_1619 | PTS system, mannitol-specific IIA component |
| LSL_1628 | Transcription regulator |
| LSL_1629 | Na+/H+ antiporter |
| LSL_1644 | Branched-chain amino acid transport protein |
| LSL_1648 | Phage integrase |
| LSL_1650 | Antirepressor |
| LSL_1651 | Hypothetical protein, phage associated |
| LSL_1653 | Hypothetical protein, phage associated |
| LSL_1656 | Hypothetical protein, phage associated |
| LSL_1657 | Hypothetical protein, phage associated |
| LSL_1658 | DNA primase |
| LSL_1661 | Hypothetical protein, phage associated |
| LSL_1665 | Hypothetical protein, phage associated |
| LSL_1667 | Transcriptional regulator, TetR family |
| LSL_1668 | Hypothetical membrane spanning protein |
| LSL_1670 | D-alanine aminotransferase |

| | |
|---|---|
| LSL_1672 | Hypothetical membrane spanning protein |
| LSL_1674 | Agmatine deiminase |
| LSL_1677 | Transcriptional regulator, TetR family |
| LSL_1680 | Glyoxalase family protein |
| LSL_1681 | 2,5-diketo-D-gluconic acid reductase |
| LSL_1686 | Thioredoxin peroxidase |
| LSL_1688 | Transcriptional regulator, TetR family |
| LSL_1709 | ThiJ/PfpI family protein |
| LSL_1711 | Transcriptional regulator, LacI family |
| LSL_1714 | PTS system, mannose-specific IID component |
| LSL_1715 | PTS system, mannose-specific IIC component |
| LSL_1716 | PTS system, mannose-specific IIAB component |
| LSL_1719 | Conserved hypothetical protein |
| LSL_1720 | Hypothetical membrane spanning protein |
| LSL_1738 | LSU ribosomal protein L34P |
