# Supplementary material for: Genome sequences and comparative genomics of two Lactobacillus ruminis strains from the bovine and human intestinal tracts
Source: Microb Cell Fact. 2011 Aug 30;10(Suppl 1):S13. doi: 10.1186/1475-2859-10-S1-S13 (PMC3231920; doi:10.1186/1475-2859-10-S1-S13)
Supplement: Additional File 15 — Proteins that were common to all six Lactobacillus groups analyzed [file 1475-2859-10-S1-S13-S15.pdf]

Core proteins in all 6 lactobacillus species analyzed
1-acyl-sn-glycerol-3-phosphate acyltransferase
16S rRNA m(5)C 967 methyltransferase
16S rRNA methyltransferase GidB
16S rRNA processing protein
23S rRNA methyltransferase
23S rRNA methyltransferase
3-hydroxy-3-methylglutaryl-coenzyme A reductase/3-hydroxy-3-methylglutaryl-coenzyme Auctase
30S ribosomal protein S1
30S ribosomal protein S10
30S ribosomal protein S11
30S ribosomal protein S12
30S ribosomal protein S13
30S ribosomal protein S16
30S ribosomal protein S17
30S ribosomal protein S18
30S ribosomal protein S19
30S ribosomal protein S2
30S ribosomal protein S20
30S ribosomal protein S21
30S ribosomal protein S3
30S ribosomal protein S4
30S ribosomal protein S5
30S ribosomal protein S7
30S ribosomal protein S8
30S ribosomal protein S9
4-oxalocrotonate tautomerase
4'-phosphopantetheinyl transferase
5-formyltetrahydrofolate cyclo-ligase
5'-methylthioadenosine nucleosidase / S-adenosylhomocysteine nucleosidase
50S ribosomal protein L1
50S ribosomal protein L10
50S ribosomal protein L11
50S ribosomal protein L12P
50S ribosomal protein L13
50S ribosomal protein L14P
50S ribosomal protein L15P
50S ribosomal protein L16
50S ribosomal protein L17P
50S ribosomal protein L18
50S ribosomal protein L19
50S ribosomal protein L2
50S ribosomal protein L20
50S ribosomal protein L21
50S ribosomal protein L22
50S ribosomal protein L23P
50S ribosomal protein L24P
50S ribosomal protein L27,ABC superfamily ATP binding cassette transporter, substrate binding protein
50S ribosomal protein L28
50S ribosomal protein L29
50s ribosomal protein L3
50S ribosomal protein L30
50S ribosomal protein L31 type B

50S ribosomal protein L35
50S ribosomal protein L4
50S ribosomal protein L5
50S ribosomal protein L6P
50S ribosomal protein L7AE
50S ribosomal protein L9P
6-phosphogluconate dehydrogenase
6-phosphogluconolactonase
ABC superfamily ATP binding cassette transporter, ABC protein
ABC superfamily ATP binding cassette transporter, binding protein
ABC superfamily ATP binding cassette transporter, binding protein
ABC transport ATP-binding protein
ABC transport ATP-binding protein
ABC transport permease protein
ABC transporter permease protein
ABC transporter, ATP binding protein
ABC transporter, ATP-binding protein
ABC transporter, ATP-binding protein
ABC transporter, ATP-binding protein
ABC transporter, ATP-binding protein
ABC transporter, ATP-binding protein
Acetate kinase
acetyltransferase
acyl carrier protein
Acyl-acyl carrier protein thioesterase
Acylphosphatase
Acyltransferase
Adenine-specific methyltransferase
Adenylate kinase/nucleoside-diphosphate kinase
Adenylosuccinate lyase
Adenylosuccinate synthetase
ADP-ribose pyrophosphatase
Alanine racemase
Alanyl-tRNA synthetase
Aldehyde-alcohol dehydrogenase
aldose 1-epimerase
alkaline shock protein
Alpha-amylase
Amino acid ABC transporter, ATP-binding protein
Aminopeptidase N
Aminopeptidase/Bleomycin hydrolase
Arginyl-tRNA synthetase
asparagine synthase
asparaginyl-tRNA synthetase
aspartyl-tRNA synthetase
Aspartyl/glutamyl-tRNA(Asn/Gln) amidotransferase subunit A
Aspartyl/glutamyl-tRNA(Asn/Gln) amidotransferase subunit B
Aspartyl/glutamyl-tRNA(Asn/Gln) amidotransferase subunit C
ATP synthase B chain
ATP synthase delta chain
ATP synthase epsilon chain
ATP-binding Clp protease subunit
ATP-dependant DNA helicase
ATP-dependent Clp protease ATP-binding subunit

ATP-dependent Clp protease proteolytic subunit
ATP-dependent DNA helicase
ATP-dependent DNA helicase
ATP-dependent helicase, DinG family
ATP-dependent protease La
ATP-dependent RNA helicase
ATP-dependent RNA helicase
ATP-dependent RNA helicase
ATP-NAD kinase
ATP/GTP hydrolase
Bacterial Peptide Chain Release Factor 2
Beta-lactamase domain protein
Beta-lactamase family protein
Catabolite control protein A
Cation ABC superfamily ATP binding cassette transporter, membrane protein
cation transport ATPase
CBS domain containing protein
CDP-diacylglycerol--glycerol-3-phosphate 3-phosphatidyltransferase
cell division initiation protein DivIVA
Cell division protein
cell division protein
cell division protein
cell division protein FtsA
Cell division protein FtsH
cell division protein FtsZ
cell shape determining protein
chaperone DnaJ
chaperone DnaK
Chaperonin GroEL
Chaperonin GroES
Chloride channel protein
chromosomal replication initiation protein
chromosome partition protein
chromosome partitioning protein, DNA-binding protein
cobyric acid synthase
ComE operon protein 1
ComE operon protein 3
ComG operon protein 1
conserved Carboxymuconolactone decarboxylase family protein
conserved hypothetical membrane protein
Conserved hypothetical membrane protein
Conserved hypothetical protein
Conserved hypothetical protein
Conserved hypothetical protein
Conserved hypothetical protein
Conserved hypothetical protein
Conserved hypothetical protein
conserved hypothetical protein
conserved hypothetical protein
conserved hypothetical protein
conserved hypothetical protein
Conserved hypothetical protein
Conserved hypothetical protein
Conserved hypothetical protein

Conserved hypothetical protein
conserved hypothetical protein
conserved hypothetical protein
conserved hypothetical protein
conserved hypothetical protein
conserved hypothetical protein
conserved hypothetical protein
conserved hypothetical protein
conserved hypothetical protein
Conserved hypothetical protein
Conserved hypothetical protein
Conserved hypothetical protein
Conserved hypothetical protein
Conserved hypothetical protein
Conserved hypothetical protein
Conserved hypothetical protein
Conserved hypothetical protein
Conserved hypothetical protein, YbbR-like
Conserved hypothetical protein/Initiation-control protein
Copper chaperone
CRP family transcriptional regulator
cyclopropane-fatty-acyl-phospholipid synthase
cysteine desulfurase / selenocysteine lyase
cysteinyl-tRNA synthetase
cytidylate kinase
Cytosine/adenosine deaminase
D-alanine transfer protein DltD
D-alanine--poly(phosphoribitol) ligase subunit 1
D-alanyl carrier protein
D-fructose-6-phosphate amidotransferase
D-tyrosyl-tRNA(Tyr) deacylase
DEAD/DEAH box helicase
dedA family protein
DegV family protein
DegV family protein
Deoxyuridine 5'-triphosphate nucleotidohydrolase
Dephospho-CoA kinase
DHH family phosphoesterase
Di-/tripeptide transporter
di-trans-poly-cis-decaprenylcistransferase
Diadenosine tetraphosphate (Ap4A) hydrolase related HIT family hydrolase
Dimethyladenosine transferase
dipeptidase
diphosphomevalonate decarboxylase
DltB D-alanyl transfer protein
DNA gyrase subunit A
DNA gyrase subunit B
DNA mismatch repair protein
DNA mismatch repair protein
DNA mismatch repair protein MutS
DNA polymerase I
DNA polymerase III alpha subunit
DNA polymerase III PolC
DNA polymerase III subunit beta

DNA polymerase III subunit gamma/tau
DNA processing protein
DNA repair protein
DNA repair protein RadA
DNA repair protein RecN
DNA replication and repair protein
DNA topoisomerase I
DNA topoisomerase IV subunit A
DNA topoisomerase IV subunit B
DNA-3-methyladenine glycosylase
DNA-binding protein
DNA-directed DNA polymerase III, epsilon chain
DNA-directed RNA polymerase subunit alpha
DNA-directed RNA polymerase subunit beta
DNA-directed RNA polymerase subunit beta
DNA-directed RNA polymerase subunit delta
DNA-directed RNA polymerase, omega subunit
elongation factor G
elongation factor Tu
excinuclease ABC subunit A
Excinuclease ABC subunit B
Excinuclease ABC subunit C
exodeoxyribonuclease V alpha chain
exodeoxyribonuclease VII large subunit
exodeoxyribonuclease VII small subunit
F0F1 ATP synthase subunit A
F0F1 ATP synthase subunit alpha
F0F1 ATP synthase subunit beta
F0F1 ATP synthase subunit C
F0F1 ATP synthase subunit gamma
Fatty acid/phospholipid synthesis protein
Flavodoxin
folylpolyglutamate synthase / dihydrofolate synthase
Formamidopyrimidine-DNA glycosylase
formate-tetrahydrofolate ligase
Galactokinase
Galactose-1-phosphate uridylyltransferase
GatB/Yqey domain-containing protein
Geranylgeranyl pyrophosphate synthase
Glucose-6-phosphate isomerase
Glucose-6-phosphate-1-dehydrogenase
Glucose-inhibited division protein A
glutamine synthetase
glutamyl-tRNA synthetase
Glyceraldehyde-3-phosphate dehydrogenase
Glycerol uptake facilitator protein
Glycerol-3-phosphate dehydrogenase (NAD(P)+)
Glycoprotein endopeptidase
Glycosyltransferase
Glycosyltransferase
Glycosyltransferase
Glycyl-tRNA synthetase alpha chain
Glycyl-tRNA synthetase beta chain
GntR family transcriptional regulator

GrpE protein
GTP pyrophosphokinase
GTP-binding protein
GTP-binding protein
GTP-binding protein
GTP-binding protein EngA
GTP-binding protein Era
GTP-binding protein LepA
GTP-binding protein YqeH
GTP-binding protein, GTPase
GTP-binding protein, probable translation factor
GTPase
GTPase ObgE
HAD superfamily hydrolase
HD superfamily hydrolase
Heat shock protein
heat-inducible transcription repressor
Hemolysin III
histidyl-tRNA synthetase
Holliday junction DNA helicase
Holliday junction DNA helicase RuvA
HPr kinase/phosphorylase
Hydrolase
Hydroxymethylglutaryl-CoA synthase
Hypothetical membrance protein
Hypothetical membrane protein
Hypothetical membrane protein
Hypothetical membrane protein
Hypothetical membrane protein
Hypothetical protein
hypothetical protein
Hypoxanthine-guanine phosphoribosyltransferase
Iojap-related protein
isochorismatase
isoleucyl-tRNA synthetase
isopentenyl pyrophosphate isomerase
L-lactate dehydrogenase
leucyl-tRNA synthetase
Lysyl-tRNA synthetase
magnesium and cobalt efflux protein CorC
manganese-dependent inorganic pyrophosphatase
MecA-type protein
Mechanosensitive ion channel
metallo-beta-lactamase superfamily protein
metallo-beta-lactamase superfamily protein
metalloprotease
Methionine aminopeptidase
methionyl-tRNA formyltransferase
Methionyl-tRNA synthetase
methylenetetrahydrofolate dehydrogenase (NADP+) / methenyltetrahydrofolate cyclohydrolase
Methyltransferase
methyltransferase
Methyltransferase superfamily protein
MFS family major facilitator transporter

Multidrug resistance ABC transporter ATP-binding and permease protein
multimodular transpeptidase-transglycosylase PBP 1A
Muramidase
N-acetylglucosamine catabolic protein
Na+/H+ antiporter
NAD synthetase
NAD-dependent DNA ligase
Nicotinate-nucleotide adenylyltransferase
Nitroreductase
nucleic acid binding protein
O-sialoglycoprotein endopeptidase
Oligoendopeptidase O
orotate phosphoribosyltransferase
orotidine 5'-phosphate decarboxylase
Oxidoreductase
Pantothenate kinase
penicillin binding protein
penicillin-binding protein
peptide chain release factor 1
Peptide Chain Release Factor 3
peptide deformylase
peptide methionine sulfoxide reductase
peptide release factor-glutamine N5-methyltransferase
peptidyl-prolyl cis-trans isomerase
Peptidyl-tRNA hydrolase
Peptidylprolyl isomerase
phenylalanyl-tRNA synthetase subunit alpha
phenylalanyl-tRNA synthetase subunit beta
Phosphate ABC transporter permease
Phosphate ABC transporter permease
Phosphate acetyltransferase
phosphate starvation-inducible protein
Phosphate transporter PhoU
phosphatidate cytidylyltransferase
phospho-N-acetylmuramoyl-pentapeptide-transferase
Phosphocarrier protein HPr
Phosphodiesterase
Phosphoenolpyruvate-protein phosphotransferase
Phosphoesterase, DHH family protein
Phosphoglucomutase
phosphoglucosamine mutase
Phosphoglycerate kinase
phosphoglycerate mutase
phosphoglycerate mutase
Phosphoketolase
phosphomevalonate kinase
phosphopantetheine adenylyltransferase
phosphopantothenoylcysteine decarboxylase / phosphopantothenate-cysteine ligase
Phosphopyruvate hydratase
polar amino acid ABC transporter permease
polar amino acid ABC transporter substrate-binding protein
Poly(A) polymerase / tRNA nucleotidyltransferase
Possible endopeptidase
possible protein-tyrosine-phosphatase

Predicted nucleic-acid-binding protein implicated in transcription termination
Predicted rRNA methylase
Preprotein translocase subunit
Preprotein translocase subunit SecY
Preprotein translocase subunit YidC
Primase
Primosomal protein DnaI
primosomal protein n'
Prolipoprotein diacylglyceryl transferase
prolyl-tRNA synthetase
protein phosphatase 2C
Protein Translation Elongation Factor Ts
Protein translocase subunit SecA
Protein translocase subunit SecE
pseudouridine synthase
Pur operon repressor
putative fibronectin-binding protein
Putative nucleic acid methyltransferase
Pyruvate kinase
Recombinase A
Recombination protein RecR
recombination protein RecU
Recombination regulator RecX
Redox-sensitive transcriptional regulator Rex
RelA/SpoT domain-containing protein
Replicative DNA helicase
rhomboid family integral membrane protein
Riboflavin kinase
Ribokinase
Ribonuclease BN
ribonuclease HII
Ribonuclease III
ribonuclease III
Ribonuclease M5
Ribonuclease P
Ribonuclease R 1
Ribonuclease Z
Ribonucleotide-diphosphate reductase subunit beta
Ribose-5-phosphate isomerase A
Ribose-phosphate pyrophosphokinase
ribosomal large subunit pseudouridine synthase
ribosomal protein S6
Ribosome-associated factor Y
ribosome-binding factor A
RibT protein
ribulose-phosphate 3-epimerase
RNA polymerase sigma factor RpoD
rod shape determining protein
rod shape-determining protein
rod shape-determining protein MreB
S-adenosyl-methyltransferase
S-adenosylmethionine synthetase
S4 domain protein YaaA
segregation and condensation protein A

segregation and condensation protein B
Seryl-tRNA synthetase
Short chain dehydrogenase
Signal peptidase I
signal peptidase II
Signal recognition particle associated protein
single stranded binding protein
site-specific recombinase XerD
Sortase
Spermidine/putrescine ABC transporter
SsrA-binding protein
SSU ribosomal protein S15P
Stage III sporulation protein J
Sua5/YciO/YrdC/YwlC family protein
Sugar kinase
Sugar transport
Sulfatase family protein
TatD family deoxyribonuclease
TerC faamily membrane protein
Tetrapyrrole (Corrin/Porphyrin) methylase family protein
Tetratricopeptide repeat family protein
thiamin pyrophosphokinase
thiamine biosynthesis protein
Thioredoxin
Thioredoxin
Thioredoxin reductase
Thioredoxin reductase
Threonyl-tRNA synthetase
Thymidylate kinase
thymidylate synthase
Transcription antitermination protein
transcription elongation factor
transcription elongation factor NusA
transcription regulator
Transcription-repair coupling factor
Transcriptional regulator
transcriptional regulator
Transcriptional regulator NrdR
Transcriptional regulator Spx
translation initiation factor IF-1
Translation initiation factor IF-3
Transmembrane histidine kinase
trigger factor, PPIase
Triosephosphate isomerase
tRNA (5-methylaminomethyl-2-thiouridylate)-methyltransferase
tRNA (guanine-N(1)-)-methyltransferase
tRNA (m(7)G46) methyltransferase
tRNA (Uracil-5-) -methyltransferase
tRNA (Uracil-5-) -methyltransferase
tRNA binding domain protein
tRNA delta(2)-isopentenylpyrophosphate transferase
tRNA modification GTPase TrmE
tRNA pseudouridine synthase
tRNA pseudouridine synthase B

tRNA-dihydrouridine synthase
tRNA/rRNA methyltransferase
Tryptophanyl-tRNA synthetase II
Two component system histidine kinase
Two component system histidine kinase
Two-component response regulator
Two-component response regulator
two-component response regulator
Two-component response regulator
Two-component sensor kinase
Tyrosyl-tRNA synthetase
UDP-glucose 4-epimerase
UDP-N-acetylenolpyruvoylglucosamine reductase
UDP-N-acetylglucosamine 1-carboxyvinyltransferase
UDP-N-acetylmuramate--L-alanine ligase
UDP-N-acetylmuramoyl-L-alanyl-D-glutamate synthetase
UDP-N-acetylmuramoyl-tripeptide--D-alanyl-D-alanine ligase
UDP-N-acetylmuramyl tripeptide synthase (putative)
undecaprenyldiphospho-muramoylpentapeptide beta-N-acetylglucosaminyltransferase
universal stress protein
uracil phosphoribosyltransferase
uracil phosphoribosyltransferase
Uracil-DNA glycosylase
uridine kinase
uridylate kinase
UTP--glucose-1-phosphate uridylyltransferase
valyl-tRNA synthetase
Xaa-His dipeptidase
Xaa-Pro aminopeptidase
Xaa-Pro dipeptidyl-peptidase
Xanthine permease
Xanthine phosphoribosyltransferase
Xanthine uracil permease
