# Supplementary material for: Genome sequences and comparative genomics of two Lactobacillus ruminis strains from the bovine and human intestinal tracts
Source: Microb Cell Fact. 2011 Aug 30;10(Suppl 1):S13. doi: 10.1186/1475-2859-10-S1-S13 (PMC3231920; doi:10.1186/1475-2859-10-S1-S13)
Supplement: Additional File 16 — Proteins unique to six lactobacillus groups relative to the combined protein set of all other species in the analysis [file 1475-2859-10-S1-S13-S16.pdf]

Unique proteins by lactobacillus Group

Group A

response regulator
hypothetical protein
ATP-dependent helicase
ABC transporter ATP binding and permease protein
hypothetical protein
amino acid permease
putative cell shape determining protein
chromosome replication initiation
hypothetical protein
cell division protein
ribose operon repressor
hypothetical protein
hypothetical protein
transketolase, beta subunit
pyridine mercuric reductase
ATP-dependent exonuclease subunit B
putative multidrug efflux permease
2-deoxyribosyltransferase
hypothetical protein
putative ribose operon repressor
hypothetical protein
cation transporter P-ATPase
putative HAD superfamily hydrolase
hypothetical protein
hypothetical protein
alkylphosphonate ABC transporter
glycosyl transferase
alkaline phosphatase
transcriptional regulator, glucose kinase
putative glycosyl transferase
hypothetical protein
sucrose phosphorylase
cell division regulator
ABC transporter ATPase and permease protein
putative glucan mod. protein

Group B

chloride channel protein
PTS system galactitol-specific IIC component
transport protein
hypothetical protein
hypothetical protein
putative transport protein

Group C

Beta-galactosidase/beta-glucuronidase
2-dehydro-3-deoxygluconokinase

hypothetical protein
permease of the major facilitator superfamily
cAMP-binding protein - catabolite gene activator and regulatory subunit of cAMP-dependent protein kinase
transcriptional regulator
Amino acid transporter
Na+/xyloside symporter related transporter
Predicted membrane protein

Group D

transcription regulator
purine transport regulator
2-keto-3-deoxygluconate kinase
transport protein
teichoic acid biosynthesis protein
phosphonates ABC transporter, permease protein
oxidoreductase
cell surface hydrolase, membrane-bound (putative)
transport protein
citrate lyase regulator
transcription regulator
unknown
sulfate adenylyltransferase
transcription regulator
transcription regulator
unknown
UDP-N-acetylmuramoylalanyl-D-glutamate--2, 6-diaminopimelate ligase
phosphoglycolate phosphatase
ABC transporter, substrate binding protein
transcription regulator
unknown
(2-aminoethyl)phosphonate--pyruvate aminotransferase
ABC transporter, ATP-binding protein
response regulator PlnC, activator
glycosyltransferase
myo-inositol 2-dehydrogenase
prophage Lp2 protein 2, integrase
transcription regulator
glycosyltransferase
purine/pyrimidine phosphoribosyltransferase (putative)
unknown
cobalt transport protein
transcription regulator
transcription regulator (putative)
3-phosphoshikimate 1-carboxyvinyltransferase
transcription regulator
transcription regulator
transport protein
integrase/recombinase, fragment (putative)
histidine protein kinase; sensor protein
glycosyltransferase
prophage Lp1 protein 52, endolysin
histidinol-phosphate aminotransferase

histidine--tRNA ligase (putative)
amino acid transport protein
glycosyltransferase (rhamnosyltransferase)
nitrate ABC transporter, permease protein
unknown
unknown
transcription regulator
DegV family protein
transport protein
transport protein
bifunctional protein-glutamate kinase; acetyl-ornithine deacetylase (putative) 435955:437793 forward
myo-inositol 2-dehydrogenase
transcription regulator
transcription regulator
cell surface hydrolase
cell surface protein precursor
poly(glycerol-phosphate) alpha-glucosyltransferase
rhamnulokinase
sugar kinase and transcription regulator
2-haloacid dehalogenase (putative)
transcription regulator
glycosyltransferase
transcription regulator
transcription regulator
non-ribosomal peptide synthetase NpsB
glucose-1-phosphate adenylyltransferase, subunit
5-methyltetrahydropteroyltriglutamate-- homocysteine S-methyltransferase
extracellular protein
transport protein
transcription regulator
aminotransferase with N-terminal regulator domain
oxidoreductase
unknown
glycosyltransferase

Group E

Amino acid ABC transporter, substrate binding protein
marR family Transcriptional regulator
Cell division protein FtsL
Hypothetical membrane protein
Chromosome replication initiation / membrane attachment protein
DNA topoisomerase III
Conserved hypothetical protein
oligoendopeptidase F
Hypothetical membrane protein

Group X

hypothetical protein
Predicted phosphatase
transcriptional antiterminator, BglG family
aminotransferase
permease of the major facilitator superfamily

Uncharacterized membrane-bound protein conserved in bacteria
Nucleotidyltransferase/DNA polymerase for DNA repair
pyruvate phosphate dikinase
hypothetical protein
permease of the major facilitator superfamily
