# Supplementary material for: Genome sequences and comparative genomics of two Lactobacillus ruminis strains from the bovine and human intestinal tracts
Source: Microb Cell Fact. 2011 Aug 30;10(Suppl 1):S13. doi: 10.1186/1475-2859-10-S1-S13 (PMC3231920; doi:10.1186/1475-2859-10-S1-S13)
Supplement: Additional File 5 — Pyruvate metabolism of L. ruminis ATCC 27782. Enzyme labels in green boxes represent those for which the corresponding gene was annotated in the genome. [file 1475-2859-10-S1-S13-S5.pdf]

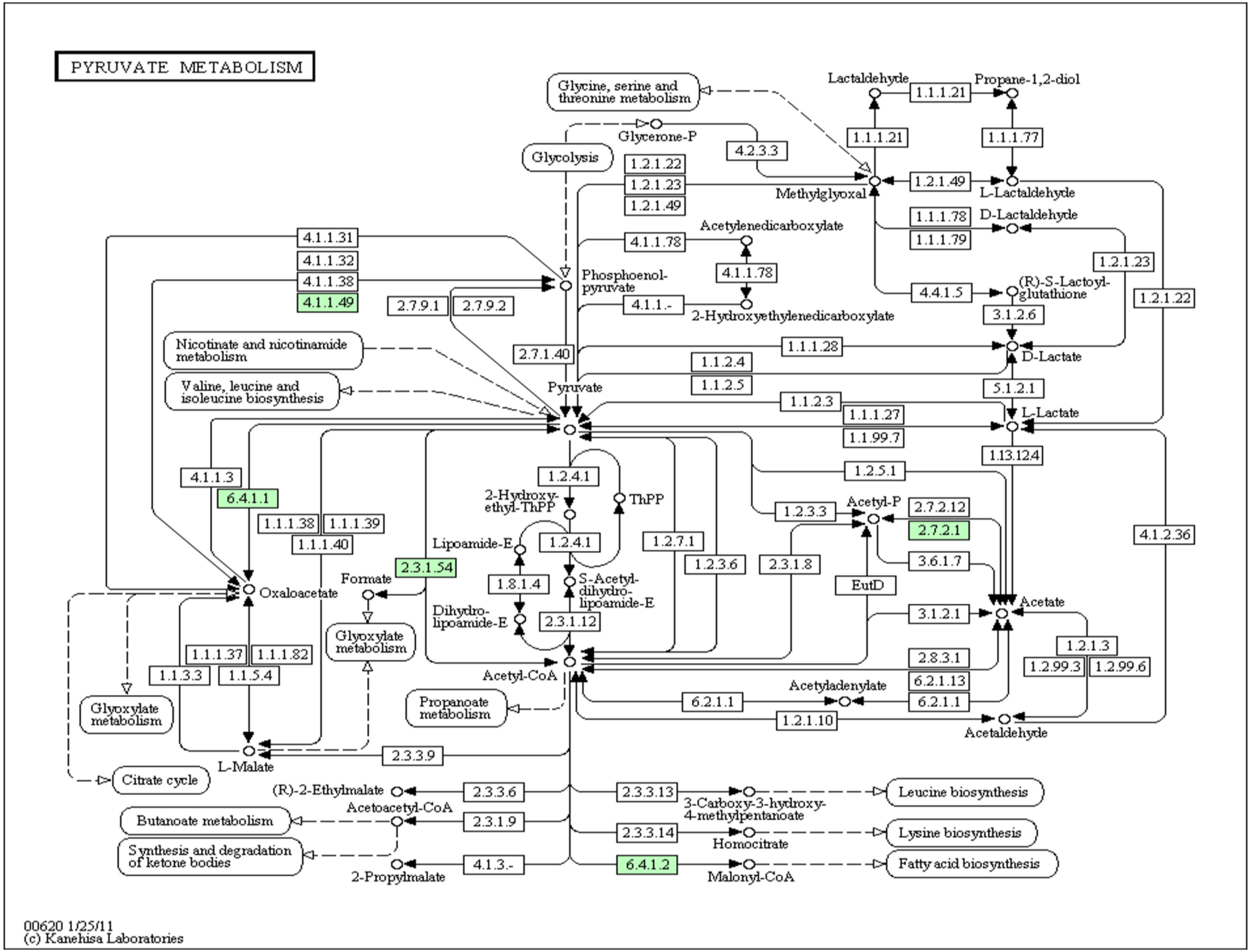
PYRUVATE METABOLISM
Glycine, serine and threonine metabolism
Glycolysis
Glycerone-P
4.2.3.3
1.2.1.22
1.2.1.23
1.2.1.49
Methylglyoxal
Lactaldehyde
1.1.1.21
Propane-1,2-diol
1.1.1.21
1.1.1.77
1.2.1.49
L-Lactaldehyde
1.1.1.78
1.1.1.79
D-Lactaldehyde
1.2.1.23
4.4.1.5
(R)-S-Lactoyl-glutathione
3.1.2.6
1.2.1.22
4.1.1.31
4.1.1.32
4.1.1.38
4.1.1.49
4.1.1.78
Acetylenedicarboxylate
4.1.1.78
4.1.1.-
2-Hydroxyethylenedicarboxylate
Phosphoenol-pyruvate
2.7.9.1
2.7.9.2
2.7.1.40
1.1.1.28
D-Lactate
1.1.2.4
1.1.2.5
5.1.2.1
Nicotinate and nicotinamide metabolism
Valine, leucine and isoleucine biosynthesis
Pyruvate
1.1.2.3
1.1.1.27
1.1.99.7
L-Lactate
1.13.12.4
1.2.5.1
4.1.1.3
6.4.1.1
1.1.1.38
1.1.1.39
1.1.1.40
1.2.4.1
2-Hydroxy-ethyl-ThPP
ThPP
1.2.4.1
Lipoamide-E
1.8.1.4
S-Acetyl-dihydro-lipoamide-E
Dihydro-lipoamide-E
2.3.1.12
2.3.1.54
Formate
Glyoxylate metabolism
1.2.7.1
1.2.3.6
2.3.1.8
1.2.3.3
Acetyl-P
2.7.2.12
2.7.2.1
3.6.1.7
EutD
4.1.2.36
Acetate
3.1.2.1
2.8.3.1
6.2.1.13
6.2.1.1
1.2.1.3
1.2.99.3
1.2.99.6
Oxaloacetate
1.1.1.37
1.1.1.82
1.1.3.3
1.1.5.4
Glyoxylate metabolism
Acetyl-CoA
Propanoate metabolism
Acetyladenylate
6.2.1.1
1.2.1.10
Acetaldehyde
L-Malate
2.3.3.9
Citrate cycle
(R)-2-Ethylmalate
2.3.3.6
Acetoacetyl-CoA
2.3.1.9
Butanoate metabolism
Synthesis and degradation of ketone bodies
2-Propylmalate
4.1.3.-
2.3.3.13
3-Carboxy-3-hydroxy-4-methylpentanoate
Leucine biosynthesis
2.3.3.14
Homocitrate
Lysine biosynthesis
6.4.1.2
Malonyl-CoA
Fatty acid biosynthesis
00620 1/25/11
(c) Kanehisa Laboratories
